# Supplementary material for: The use of humanure for cereal production under conventional and regenerative farming models - findings from a three-year grassland-to-arable transition
Source: PLoS One. 2026 Mar 6;21(3):e0335625. doi: 10.1371/journal.pone.0335625 (PMC12965554; doi:10.1371/journal.pone.0335625)
Supplement: S1 Table — (DOCX) [file pone.0335625.s001.docx]

**S1 Table. Detailed Plot Management Diary.**

| Year | Date | Conventional Plots | Regenerative Plots | Comments |
| --- | --- | --- | --- | --- |
| Year 1 | 01/06/2021 | Entire area ploughed with reversible, fixed width plough. | |  |
|  | 15/06/2021 | Entire area treated with Motif herbicide (MAPP 165509) from backpack sprayer at 5.0 L/ha | |  |
|  | 02/08/2021 | Entire area treated with Motif herbicide (MAPP 165509) from backpack sprayer at 5.0 L/ha | |  |
|  | 21/09/2021 | Primary and secondary power-harrow with Alpego 2m harrow | |  |
|  | 23/09/2021 | Baseline soil samples taken | |  |
|  | 24/09/2021 | Winter wheat (Syngenta Gleam) sown with a 1m carousel trial drill with Suffolk coulters. Sowing rate of 200kg/ha | Winter wheat (Syngenta Gleam) sown with a 1m carousel trial drill with Suffolk coulters. Sowing rate of 200kg/ha, with Red clover (Avisto) at 13kg/ha |  |
|  | 24/09/2021 | All plots rolled with Cambridge roller | |  |
|  | 01/10/2021 | Slug pellets applied (Sluxx HP, MAPP 16571) using spreader. | |  |
|  | 08/10/2021 | Pre-emergence herbicides (Crystal MAPP 13914 and Hurricane MAPP 16027) applied at 4L/ha and 0.1L/ha respectively using Azo backpack sprayer. |  |  |
|  | 08/11/2021 | Slug pellets applied (Sluxx HP, MAPP 16571) using spreader. | | Significant pest damage (deer trampling) in October/November |
|  | 18/02/2022 |  | | Storms Eunice and Franklin waterlogging south side of the trial |
|  | 07/03/2022 | Slug pellets applied (Sluxx HP, MAPP 16571) using spreader. Herbicide Motif (MAPP 165509) applied between plots using backpack sprayer to clearly mark boundaries. | |  |
|  | 14/03/2022 | Humanure and SF applied by hand at 50kgN/ha to relevant plots. | |  |
|  | 29/03/2022 | SF mis-applied at 50kgN/ha |  |  |
|  | 31/03/2022 |  | SF applied at 50kgN/ha to match conventional plots. |  |
|  | 11/04/2022 | T0 Fungicide (Tebucon 250 EW, MAPP 17823) applied at 0.5L/ha using Azo backpack sprayer. PGR (Transit & Moddus, MAPP 15151) applied at 2L/ha and 0.1L/ha respectively using Azo backpack sprayer. |  |  |
|  | 20/04/2022 | Humanure and SF applied by hand at 100kgN/ha to relevant plots. | |  |
|  | 05/05/2022 | T1 Fungicides applied with Azo sprayer. Reyystar XE (MAPP 19250) applied at 1L/ha. Comet 200 (MAPP 12639) applied at 0.4L/ha. |  |  |
|  | 13/05/2022 | Humanure and SF applied by hand at 100kgN/ha to relevant plots. | |  |
|  | 27/05/2022 | T2 Fungicide (Univoq, MAPP 19930) applied at 1.25L/ha using Azo backpack sprayer. |  |  |
|  | 13/06/2022 | T3 Fungicide (Prosaro, MAPP 16732) applied at 0.6L/ha using Azo sprayer. |  |  |
|  | 27/07/2022 | Plant samples collected | |  |
|  | 11/08/2022 | Wheat harvested with Sampo Roseline 2010 combine harvester. | | Drought and heatwave throughout summer and harvest time |
| Year 2 | 25/08/2022 |  | | First significant rainfall in months. |
|  | 05/09/2022 |  | Fodder radish drilled with grass drill at sowing rate of 15kg/ha. Rolled with Cambridge roller |  |
|  | 07/09/2022 |  | Slug pellets applied (Ironflexx, MAPP 18555) at 7kg/ha using ICL hand-held sprayer. |  |
|  |  |  |  | Exceptionally dry February. |
|  | 05/04/2023 | Spring barley (LG Diablo) drilled with Lemkin Disc drill, 1.5m width Suffolk coulters. 11 rows at 0.15m spacing, Seeded at 325 seeds/m^2^ | |  |
|  | 04/05/2023 |  |  | Poor emergence in the regen plots due to dense cover competition. Plan changed to herbal leys. |
|  | 19/05/2023 | Humanure and SF applied at 140kgN/ha. | |  |
|  | 26/05/2023 | Post-emergence herbicide (Zypar, batch number F469K4G203) applied at 0.75L/ha using Azo sprayer. |  |  |
|  | 01/08/2023 | Plant samples collected. |  |  |
|  | 12/08/2023 | Barley harvested with Sampo Roseline 2010 combine harvester. |  |  |
|  | 21/08/2023 | Soil samples collected. | |  |
| Year 3 | 01/11/2023 | Deep plough to 8 inches with reversible, fixed width plough to break down clods. |  |  |
|  | 01/11/2023 | Power harrowed and rolled with a crumble roller (does the harrowing and rolling together). | |  |
|  | 21/11/2023 | Attempted to plant winter beans (Vespa) with John Deere R6215 and a custom direct drill using Suffolk colters. 32 seeds/m^2^ for a seed rate of 218kg/ha, to a depth of 7.5cm. Conditions were too wet and soil was saturated. | | Incredibly wet autumn and winter, so a winter plant became impossible |
|  | 18/03/2024 | Pre-sow humanure applied to surface by hand at 130kgN/ha | |  |
|  | 17/04/2024 | Power harrowed (Alpego 2m harrow), drilled (Wintersteiger toolcarrier seed drill on lenkin disc coulters) and rolled (Cambridge roller). Seed was Spring barley (Laureate), sown at 205kg/ha rate. | |  |
|  | 03/05/2024 | T1 Fungicide (Siltra Xpro, MAPP 15082) applied at 1L/ha with Azo sprayer. |  |  |
|  | 23/05/2024 | Fungicides applied with Azo sprayer. Siltra Xpro (MAPP 15082) applied at 1L/ha, and Proline (MAPP 14790) applied at 0.72L/ha. | |  |
|  | 20/06/2024 |  | | Some crow damage noticed; flappers erected. |
|  | 20/08/2024 | Plant samples collected | |  |
|  | 21/08/2024 | Barley harvested with Sampo Roseline 2010 combine harvester. | |  |
|  | 03/10/2024 | Soil samples collected | |  |
|  | 06/10/2024 | Worm sampling conducted | |  |
