# Supplementary material for: The use of humanure for cereal production under conventional and regenerative farming models - findings from a three-year grassland-to-arable transition
Source: PLoS One. 2026 Mar 6;21(3):e0335625. doi: 10.1371/journal.pone.0335625 (PMC12965554; doi:10.1371/journal.pone.0335625)
Supplement: S1 Appendix — (DOCX) [file pone.0335625.s002.docx]

**S1 Appendix. Humanure batch collection and sampling.**

**Batches**

Batch 1 – 455kg of humanure were collected from the Centre for Alternative Technology, Powys on the 21st July 2021. Samples were taken from 4 different ‘bins’ on the site. These were the material which had been collected from the vaults of several long drop toilets from around the site, into which feces, urine, toilet paper and sawdust were discarded. The material had been stored at am ambient temperature for at least 2 years, and so acceptable pathogen die-off is expected (WHO, 2006). The material was collected by shovelling into rubble sacks and transporting by car. It was left in the rubble sacks to further mature for 8 months before it was mixed in a wheelbarrow and applied to the fields on 14th March and 20th April 2022.

Batch 2 – 285kg of humanure were collected from Howsham Mill, North Yorkshire on 21st January 2023. This material was excavated from an abandoned pit toilet, and an IBC tank long-drop toilet. These toilets collected feces, urine, toilet paper and sawdust, but also contained notably more discarded solid waste items, including plastic water bottles, items of clothing and menstrual hygiene products and packaging. The material was shovelled into rubble sacks and transported by car, before being emptied into a covered IBC tank for thorough mixing and left to mature for a further 4 months. During this time a storm dislodged the cover and flooded the tank, and so the material was shovelled out onto a tarpaulin to dry out and drainage holes were drilled into the tank. The material was applied to the field on 19th May.

Batch 3 – 320kg of humanure was collected from Kirkstall Valley Farm, Leeds on 12th February 2023, from a Housing Cooperative in Leeds on the 1st March 2024 and from Living Potential Care Farm CIC, Wetherby, on 7th March 2024, all in West Yorkshire. The former was stored in rubble sacks until Batch 2 had been applied to the fields, and then was emptied into the IBC tank for maturation in June 2023. The other batches were added and mixed in when they had been collected.

**Sampling**

Humanure samples were taken from each batch prior to application to determine the chemical properties to inform the application rate. Samples were collected in plastic freezer bags and transported immediately to the lab. Samples were sieved to 8mm to remove large debris. Half the material was stored fresh at 4^o^C, whilst the other was air dried. Fresh analysis was conducted within 2 weeks of the sampling date, whilst air-dry analysis was conducted within 1 month.
