# Supplementary material for: The use of humanure for cereal production under conventional and regenerative farming models - findings from a three-year grassland-to-arable transition
Source: PLoS One. 2026 Mar 6;21(3):e0335625. doi: 10.1371/journal.pone.0335625 (PMC12965554; doi:10.1371/journal.pone.0335625)
Supplement: S2 Table — (DOCX) [file pone.0335625.s003.docx]

**S2 Table. Chemical composition of the three humanure batches used in each year of the experiment.**

|  |  | Batch 1* | Batch 2 ^Ѳ^ | Batch 3^ǂ^ |
| --- | --- | --- | --- | --- |
| **pH** |  | 6.35  [5.14, 7.00] | 6.31  [6.28, 6.33] | 6.87  [6.80, 6.95] |
| **Moisture** | % of field moist substrate | 73.05  [68.12, 74.38] | 78.90  [78.43, 79.36] | 80.73  [76.27, 80.73] |
| **Organic Matter** | % of oven dry substrate | 77.67  [68.74, 82.27] | 77.04  [76.19, 77.90] | 79.61  [78.11, 80.08] |
| **Total C** | % of oven dry substrate | 35.1  [33.92, 35.78] | 30.45  [ 30.35, 30.49] | 37.83  [37.76, 37.91] |
| **Total N** | % of oven dry substrate | 2.64  [2.28, 2.98] | 1.94  [1.89, 1.94] | 2.45  [2.45, 2.47] |
| **Inorganic N** | ppm oven dry substrate | 5502  [2075, 6883] | 4069  [3560, 4579] | 2506  [2196, 5307] |
| **NH_4__N** | ppm oven dry substrate | 80.3 [29.3, 123.7] | 713.3 [639.0, 787.7] | 81.4  [56.9, 158.9] |
| **NO_3__N** | ppm oven dry substrate | 5416.8 [2045.2, 6751.4] | 3345.73 [2910.8, 3780.7] | 2558.6 [2273.3, 5135.1] |
| **C:N** |  | 13.59  [12.26, 14.88] | 15.71  [15.67, 16.07] | 15.40  [15.34, 15.47] |
| **Available P** | ppm oven dry substrate | 217 [203, 233] | 2088 [1796, 2379] | 1347 [809, 1455] |
| **K+** | ppm oven dry substrate | 4030  [898, 7246] | 16275 [15014, 17535] | - |

**n = 5; 5 separate grab samples taken from the pile. Reported as median [IQR]
^Ѳ^ n = 2; 5 grab samples compiled into one mixed bulk sample, then measured in duplicate. Reported as median [range low, range high]
^ǂ^ n = 3; 5 grab samples compiled into one mixed bulk sample, then measured in triplicate. Reported as median [range low, range high]*
