# Supplementary material for: The use of humanure for cereal production under conventional and regenerative farming models - findings from a three-year grassland-to-arable transition
Source: PLoS One. 2026 Mar 6;21(3):e0335625. doi: 10.1371/journal.pone.0335625 (PMC12965554; doi:10.1371/journal.pone.0335625)
Supplement: S2 Appendix — (DOCX) [file pone.0335625.s004.docx]

**S2 Appendix. Detailed lab methods.**

**Bulk Density**

Bulk density samples were taken in the field using a soil corer with cutting shoe. The internal cylindrical rings were 5cm diameter and 5cm height (volume 98.17cm^3^). These were collected at a depth of 5-10cm, by removing the first 5cm of topsoil before sampling.

**Procedure**

1. Oven dry samples at 105^o^C for 12 hours.
2. Weigh oven-dried sample.
3. Calculate bulk density by dividing oven-dry mass by cylinder volume.

**Water Stable Aggregates**

The Slake test determines the strength of the biological ‘glues’ binding soil together, and thus its resistance to falling apart when submerged in water.

**Procedure**

1. Sieve air-dry soil between a 2mm and 1mm sieve, to retain material between these size fractions. The larger sieve removes roots and stones whilst the 1mm sieve retains aggregates larger than 1mm in size.
2. Transfer approximately 10g of soil retained on the 1mm sieve into a pre-weighed foil container, and record the exact weight.
3. Transfer this soil carefully back to the 1mm sieve.
4. Submerge this sieve gently into a basin of shallow water, such that the water covers the soil, but does not cover the top of the sieve, which could allow soil particles to float out of the top of the sieve.
5. Start a timer and leave the sieve submerged for 5 minutes, allowing particles to slake off and fall through the sieve into the basin.
6. After 5 minutes, agitate the soil by lifting and re-submerging the sieve in and out of the water 5 times with a brisk motion. Take care that water does not go over the top of the sieve. This will wash any more dislodged particles through the sieve into the basin**.**
7. Remove the sieve from the basin, and use a squirt bottle of water to backwash the retained soil into the pre-weighed foil container.
8. Leave on a drying rack, or heat in the oven at a low temperature (40^o^C), until the water is evaporated. This point is reached when a stable weight is achieved.
9. Reweigh the container to determine the mass of the soil which was retained on the sieve, and therefore resistant to slaking.
10. Calculate the water stable aggregates as a % of the total soil mass using the following equation:

*WSA (%) = (soil mass retained on sieve/starting soil mass) x 100*

**pH**

pH was analysed using an Oakton® pH 700 Benchtop pH Meter

**Procedure**

1. Weigh 10 ± 0.1 g air dry soil, sieved to <2mm, into a disposable plastic cup.
2. Add 25 mL of deionised water from a measuring cylinder.
3. Stir thoroughly with a plastic stirrer.
4. Stand for 15 minutes, stirring intermittently.
5. Whilst samples are ‘standing’, calibrate the electrode as per manufacturer’s instructions.
6. Stir the suspension once more with the plastic stirrer.  Insert the electrode and swirl gently in the suspension.  Make sure that the electrode is not resting on the base of the cup. Record the pH when the measurement has stabilised pH.
7. Rinse the electrode with deionised water before moving on the next sample.
8. Check the electrode stability every 10 samples with a standard buffer solution and recalibrate as needed.  Take particular care to rinse the electrode well following immersion in the buffer solution.

**Moisture Content**

Moisture content is the mass of water expressed as a percentage of the mass of the solid phase after drying in an oven at 105^o^C.

**Procedure**

1. The sample should not be sieved before weighing, but large stones and roots should be removed.
2. Record the weight of the crucible **(W1)** using a 4 figure balance.
3. Fill the crucible no more than ^3^/_4_ full with field-moist soil and then record the weight of the crucible and soil **(W2)**.
4. Place the crucibles on a metal tray and then place tray into an oven set to 105°C.
5. After 12 hours transfer the crucibles from the oven directly to a desiccator.
6. Once cool (leave for a few hours) take the samples out of the desiccator one at a time and reweigh, record the weight of the crucible and the dried soil **(W3)**.
7. Calculate moisture content (% of fresh soil) using the following equation:

*Moisture content (%) = ((fresh soil mass – dry soil mass) / fresh soil mass ) x 100*

*Moisture content (%) = (((W2-W1)-(W3-W1)) / (W3-W1)) x 100*

**Soil Organic Matter (Loss on Ignition)**

Total soil organic matter (OM) consists of both living and dead material, including well decomposed, more stabilized materials. OM analysis is a measure of carbon-containing material that is, or is derived from, living organisms, including plants and other soil dwelling organisms. Organic matter content is often provided by soil analysis laboratories along with major and minor nutrient contents, using a variety of methods.

When an oven-dry soil is heated to >450^o^C the organic matter is burnt off along with interstitial water which is not removed by air or oven drying. The mass lost is a commonly used indicator of organic material, with the inorganic material remaining behind as ash.

**Procedure**

1. Use the same samples which were used to determine moisture content.
2. Place the crucibles directly in a cold muffle furnace and allow the temperature to rise slowly to the desired ignition temperature of 550^o^C, and leave at this temperature for 12 hours.
3. Leave the furnace to cool sufficiently so the crucibles are safe to remove, and transfer directly to a desiccator.
4. Leave for a few hours to cool to room temperature, then reweigh **(W4)**.
5. Calculate the Soil Organic Matter (% of oven dried soil) using the following equation:

*SOM (%) = ((soil mass before furnace – soil mass after furnace) / soil mass before furnace) x 100*

*SOM (%) = (((W3-W1)-(W4-W1))/(W3-W1)) x 100*

**Total Elemental Carbon and Nitrogen**

A combustion elemental analyser was used to measure total Carbon and Nitrogen.

The elemental analysis is based on the high temperature combustion and subsequent analysis of the combustion gases. The quantitative separation of the analyte gases N2 and CO2 in the He carrier gas prior to the detection is crucial for the performance of the complete instrument. The analyte gases are separated on one column, by the use of separate stepped temperatures.

**Procedure**

1. Oven dry soil samples at 40 °C.
2. Grind samples to <100 µm using a Resch mixer mill MM400.
3. Check the balance is clean and level, and check the performance of the balance before, periodically and at the end of sample weighing.
4. Using tweezers, place a vario MICRO cube tin capsule on the 6 figure balance and tare the balance. Remove the tin capsule.
5. Carefully transfer soil into the tin capsule using the microspatula.
6. Return the capsule to the balance to determine if the desired weight has been obtained (~4mg), and record the exact weight on the weighing sheet.
7. Once the required amount of sample has been obtained use the tweezers to fold the parcel closed.
8. Transfer the prepared sample to a clean 96 position tray in the well that corresponds to the weighing sheet.
9. Repeat for each sample.
10. Include an appropriate CRM at the start and end of the sample set.
11. In each batch of samples include 10 % duplicates at the end of the sample set.
12. Weigh sulphanilic acid for calibration and drift standards.
13. Analyse samples using an Elementar analyser.

**Inorganic Nitrogen**

**Procedure**

**Soil preparation**

1. Sieve field-moist soil to 8mm.

**Moisture determination**

1. Carry out moisture content determination on a separate portion of field moist soil following the moisture content method.

**Extraction procedure**

1. Weigh approx 10g field moist soil into a 125 mL shaking bottle. Record the actual weight used to the nearest 0.001g.
2. All samples were repeated in triplicate, alongside 3 reagent blank samples, using the average blank value for subsequent calculations. Blank determinations are carried out by using 1M KCl without sample addition throughout the whole procedure.
3. Decant 1M KCl solution into a beaker.
4. Use a 50 mL measuring cylinder to measure 50 mL of KCl into each sample, blanks and replicate samples. Fasten the caps tightly to prevent samples leaking.
5. Shake the samples on a shaking table for 1 hour at 150 rpm.
6. Filter the sample through Whatman 42 filter paper. Discard the first few ml of filtrate, then filter the rest into pre-labelled 50 mL centrifuge tubes.
7. Centrifuge for 5 mins at 3000 rpm.
8. Decant solution into pre-labelled auto-analyser test tubes.
9. Analyse within 24 hours or freeze the sample until analysis can be carried out.  Analyse using a Skalar San++ continuous flow auto-analyser and spectrophotometric detection.

**Available Phosphorus (Olsen’s P)**

The Olsen’s P method estimates the amount of phosphorus that is potentially available for plant uptake in soils, by extraction using alkaline sodium bicarbonate (pH 8.5) solution and determining the phosphorus (P) concentration in the extract colorimetrically. It does not measure the total amount of phosphorus present in soil, since phosphorus availability is influenced by pH. This method is applicable to soils that are mildly acidic to alkaline pH and is based on the method developed by Olsen et al. (1954) to correlate crop response to fertilizer on calcareous soils.

**Procedure**

1. Air dry soil and sieve to 2mm.
2. Weigh about 2.5 g soil into a 125 mL shaking bottle.  Record the exact weight of the soil used to the nearest 0.01 g.
3. All samples were repeated in triplicate, alongside 3 reagent blank samples, using the average blank value for subsequent calculations. Blank determinations are carried out by using sodium hydrogen carbonate without sample addition throughout the whole procedure.
4. Decant the 0.5M sodium hydrogen carbonate into a beaker.
5. Use a measuring cylinder to add 50 mL of 0.5M sodium hydrogen carbonate solution to all samples, blanks and replicate samples. Fasten the caps tightly to prevent samples leaking.
6. Place on a shaker for 30 minutes at 150 rpm.
7. Within 30 minutes filter the samples through Whatman 42 filter paper. Discard the first few ml of filtrate, then filter into pre-labelled 30 mL universal containers.
8. Analyse samples using a Skalar SAN++ continuous flow auto-analyser and spectrophotometric detection.

**Exchangeable Potassium**

Exchangeable cations are those cations that are associated through electrostatic interactions with negatively charged particles in soil.  Typically, it is the organic molecules and clay particle surfaces that have negative charge.  The basic cations are the most ‘available’ to plants, and can exchange rapidly with each other and with acidic cations (Al3+ and H+), depending on the activities of each cation in soil pore waters and in percolating rainfall.

This method used ammonium chloride as an extractant, which is non-toxic and un-buffered. This means that the extractant does not artificially alter the native pH of the soil.  There is a risk that some of ammonium will be converted to nitrate in soils with pH-neutral or alkaline soils, thereby causing some acidification (Limousin & Tessier, 2003) which could affect the exchange process.  The effect is likely to be negligible for most soils and can be ignored.

**Procedure**

1. Air-dry and sieve soil to < 2 mm.
2. Weigh out approximately 2.5 g of soil into a 50 mL centrifuge tube.  Record the exact weight of soil used to the nearest 0.001 g.
3. All samples were repeated in triplicate, alongside 3 reagent blank samples, using the average blank value for subsequent calculations. Blank determinations are carried out by using sodium hydrogen carbonate without sample addition throughout the whole procedure.
4. Decant the 1M NH_4_Cl solution into a 250 mL beaker.
5. Use a 25 mL measuring cylinder to add 25 mL of 1M NH_4_Cl solution to all samples, blanks and replicates. Fasten the caps tightly to prevent samples leaking.
6. Shake on a shaker for 2 hours @ 150 rpm.
7. After shaking, centrifuge the samples for 15 minutes @ 4350 rpm.
8. Filter the samples through Whatman 42 filter paper. Discard the first few ml of filtrate, then filter into pre-labelled 50 mL centrifuge tubes.
9. Prepare a two-times dilution of the sample by pipetting 5 mL of the filtered sample into a pre-labelled15 mL centrifuge tube containing 5 mL of deionised water.  Cap the tube and shake to mix.
10. Analyse samples using ICP-OES Thermo Fisher ICAP 7600.

**Worm Count**

- Each plot was divided into thirds lengthways.
- A sample location was chosen at random from each of these thirds, using a grid and random number generator. Only the central 1m of the plot was considered for sampling due to the plots being widened in Year 2 of the experiment.
- At each sample location a 15cm^3^ hole was dug with a trowel, with all excavated material placed onto a sheet
- All the material was broken apart by hand and searched for worms.
- The total number of worms was enumerated, as well as the number of juvenile worms (measuring < 5cm length) and anecic worms.
- The worms and soil were then returned to the hole.

**Microbial Respiration**

Respiration is a measure of the metabolic activity of the soil microbial community. It is measured by capturing and quantifying carbon dioxide (CO_2_) released from a sample of soil held in an airtight jar for 1 week. Greater CO_2_ release is indicative of a larger, more active soil microbial community.


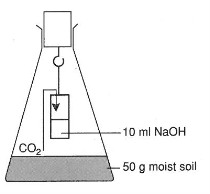


**Setup procedure**

1. Sieve field moist soil to 2mm.
2. In triplicate weigh 50 g (± 0.1) of field moist soil into respiration flasks.
3. Pipette 10 mL of 0.3M NaOH into a 10 mL tube.
4. Suspend the tube from the bung, and stopper the flask with the bung firmly.
5. Note the time each bung was placed in the flask.
6. Ensure a gas tight seal with PTFE tape.
7. Set up two control flasks in the same way but use 50 g of sand instead of the soil.
8. Store the flasks in the dark for 1 week at room temperature.
9. Determine the water content of the moist soil at the time the flasks are set up, using the moisture content method.

**Titration procedure**

1. Starting with the sand controls, remove the adhesive tape from the first conical flask. Make a note of the time the vial was removed.
2. Tip the NaOH solution from the 10 mL tube into a 250 mL conical flask. Rinse the tube with deionised water and add the rinsing water to the conical flask.
3. Pipette 10 mL of 1M BaCl_2_ solution to the conical flask.
4. Add 6 drops of phenolphthalein indicator to the conical flask and give a good swirl to mix.
5. Fill a burette with 0.1M HCl, making note of the starting volume of acid in the burette.
6. Titrate until the colour changes from pink to colourless. Once you perceive a slight change in colour, add the acid drop-by-drop until the solution is colourless.  Warning: If you add too much acid in one step you are likely to miss the end-point and underestimate the amount of CO_2_ respired.
7. Record the volume of acid remaining in the burette and calculate the total volume of acid used.
8. Tip the solution into the waste container provided and rinse out your conical flask with deionised water.
9. Repeat steps 1-8 with all samples.
10. Calculate the soil respiration rate as g CO_2_ g^-1^ air dry soil s^-1^.  The soil titration value should be between ^1^/_4_ and ^3^/_4_ of the sand control sample.  If the value is too large, errors are also large when the difference between the amount of HCl used to titrate the sample is subtracted from the amount of HCl used to titrate the sand control.  If the value is small, most of the CO_2_ has been used and the respired CO_2_ may not all have been absorbed.  The experiment may have to be repeated with the respiration time suitably adjusted.

**Calculation of results**

The volume of HCl used (ml) = Volume of acid in burette at start (ml) – volume of acid in burette at end. Call this **A**.

The amount of acid used in the experiment (in moles) = the amount of NaOH remaining (i.e. the NaOH that has **not** reacted with CO_2_. Call this **B** for the soils flask and **C** for the sand control:

**B or C** (mol) = A (~~ml~~) x ~~Litre~~ x 0.01 mol 1000 ~~ml~~ ~~Litre~~

B or C (mol) = (A x 0.01)/1000

You will find that more acid was used up for the sand control because less CO_2_ was taken up, and therefore more NaOH was available for reaction. **B and C** are the amounts of NaOH remaining in the flasks at the end of experiment, and the difference between them is the amount of NaOH that has reacted with **respired** CO_2_.

2 mol NaOH react with 1 mol CO_2_. So now we can calculate the mass (in grams) of respired CO_2_ that has reacted with NaOH. Call this **D**. The molar mass of CO_2_ is 44 g mol^-1^. Therefore the amount of respired CO_2_ which has reacted with NaOH in the flasks is:

**D** (g CO_2_) = (**C – B**) ~~mol NaOH~~ x 44 g CO_2_ x 1 ~~mol CO~~_~~2~~_

1 ~~mol CO~~_~~2~~_ 2 ~~mol NaOH~~

**D** (g CO_2_) = (**C**-**B**) x 44 x ½

Finally, it is common to report soil respiration rates on an air-dry weight basis per unit time (g CO_2_ g^-1^ dry soil s^-1^).

Oven-dry weight of incubated soil (g) = 50 g moist soil x % Dry Matter. Call this **E**.

You will also need to calculate exactly the length of time (days, hours and minutes) that your soils were incubating (see group worksheet from last week), and convert this to seconds.

Call this **F**.

Soil respiration rate (g CO_2_ g^-1^ dry soil s^-1^) = **D**_

(**E** x **F)**

**Fungal Biomass**

Fungal biomass was enumerated via direct light microscopy.

**Procedure**

1. Sieve air dry soil to 2mm
2. Using a dropping pipette, weigh the number of drops of water required to real 1ml. Use this value to calculate the volume of a single drop from that pipette.
3. Fill a 50ml centrifuge tube with 45ml of deionised water.
4. Add soil until the water is displaced to 50ml, creating a 1:10 volumetric dilution of soil, and record the dilution.
5. Cap the tube, and shake at a consistent rate 40 times with an abrupt arm motion through 90 degree swing, to encourage the breakup of soil particles.
6. Allowed to settle for 1 minute.
7. Extract solution with a pipette, and drop one drop onto a microscope slide.
8. Cover with a coverslip and record the cover slip dimensions.
9. Place the sample on the stage and setup and focus the microscope to view the sample at 400x total magnification.
10. If the sample is too dark, increase the dilution and adjust the calculations.
11.
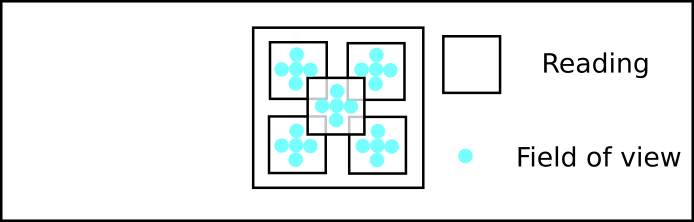
Use the following pattern to enumerate 25 fields of view (FOV).
12. In each FOV record the amount of fungi present. Use a graduated eyepiece or computer software to measure the length and diameter of each fungal filament, and record. Use the density assumption that 1 cubic cm of fungi weighs 1.5g.
13. Calculate the predicted biomass of the fungi within the entire sample, by averaging the findings of the 25 FOVs and multiplying by the total number of FOVs under the entire cover slip. This is calculated from the cover slip size, the eyepiece field size and the magnification.
14. Calculate the biomass of fungi in 1ml of soil by multiplying by the droplet number in step 2 and the dilution factor.
15. Repeat each sample in triplicate.
