# Supplementary material for: The use of humanure for cereal production under conventional and regenerative farming models - findings from a three-year grassland-to-arable transition
Source: PLoS One. 2026 Mar 6;21(3):e0335625. doi: 10.1371/journal.pone.0335625 (PMC12965554; doi:10.1371/journal.pone.0335625)
Supplement: S1 Fig — (DOCX) [file pone.0335625.s005.docx]

**S1 Fig. Barley head damage and disease examples.**


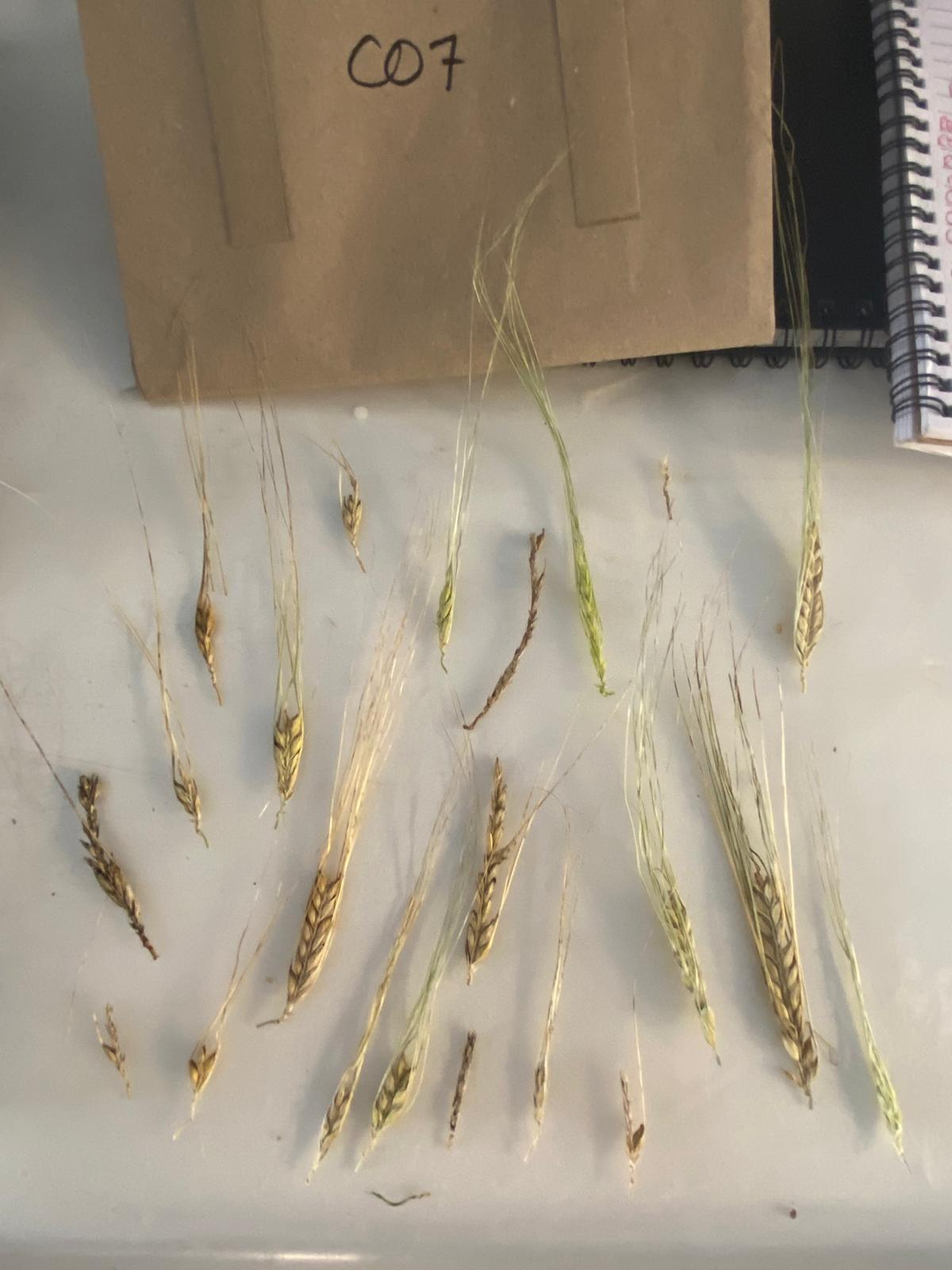


Sooty head mold

Grains missing/ not developed

Spike missing / not developed

**S1 Fig. Example of different disease and damages observed in the barley heads. In red, the grains are discoloured and have a low mass, caused by sooty head mold. In blue, central spike has grown, shown by empty rachi. The grains have either not developed or have been predated. Some were also discoloured and brittle, indicating disease. In orange, the majority of the central spike is missing or has not developed. It is not clear whether this is due to predation or disease.**
