# Supplementary material for: The use of humanure for cereal production under conventional and regenerative farming models - findings from a three-year grassland-to-arable transition
Source: PLoS One. 2026 Mar 6;21(3):e0335625. doi: 10.1371/journal.pone.0335625 (PMC12965554; doi:10.1371/journal.pone.0335625)
Supplement: S6 Fig — (DOCX) [file pone.0335625.s011.docx]

**S6 Fig. Spring barley 1000 grain mass.**


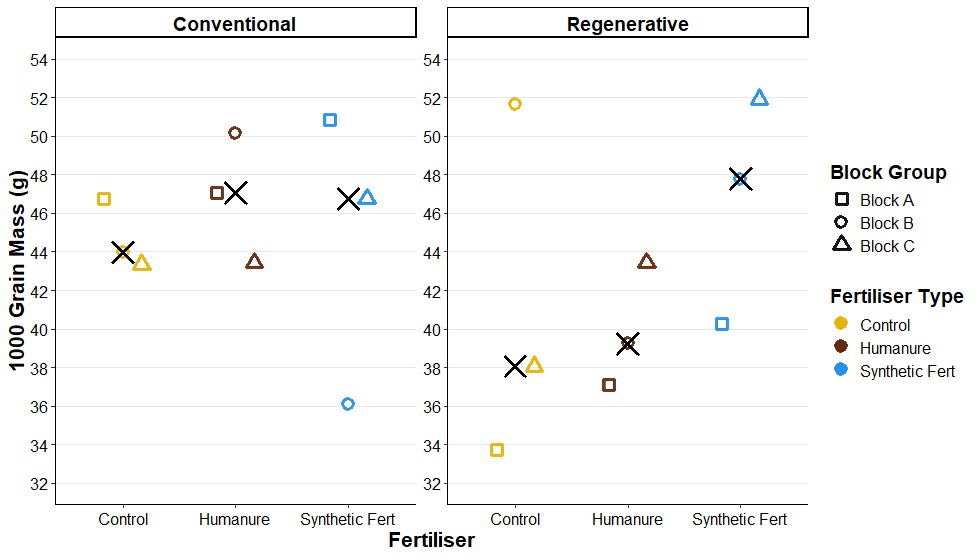

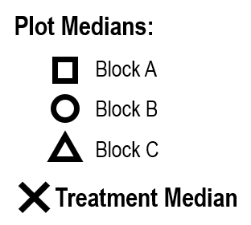


**S6 Fig. Spring barley 1000 grain mass. Data points show the mass of 1000 weighed grains.**
