# Supplementary figures and images for: The use of humanure for cereal production under conventional and regenerative farming models - findings from a three-year grassland-to-arable transition
Source: PLoS One. 2026 Mar 6;21(3):e0335625. doi: 10.1371/journal.pone.0335625 (PMC12965554; doi:10.1371/journal.pone.0335625)

**S7 Fig. Spring barley dry head biomass.**

**S7 Fig. Spring barley average dry head biomass.**


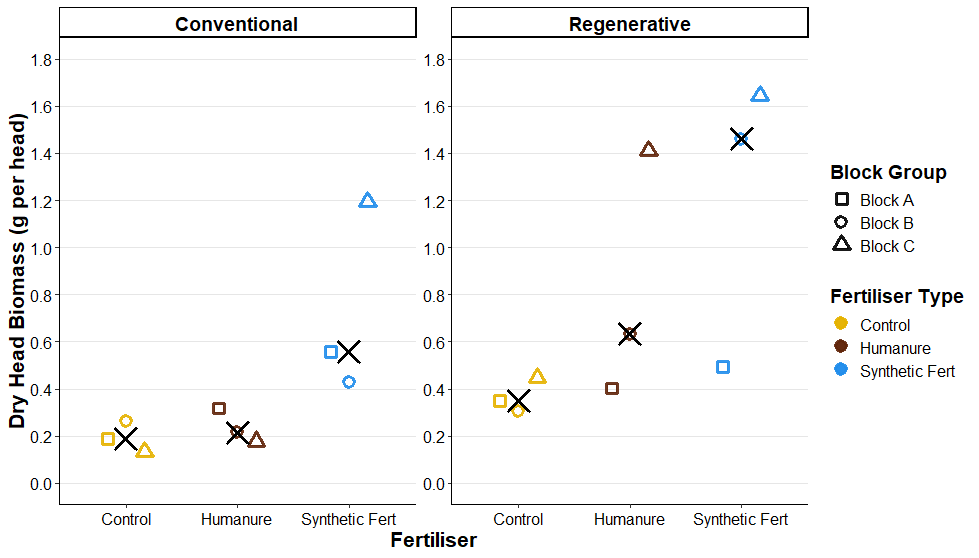

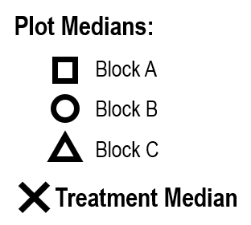

Supplement: S7 Fig — (DOCX) [file pone.0335625.s012.docx]
