# Supplementary material for: The use of humanure for cereal production under conventional and regenerative farming models - findings from a three-year grassland-to-arable transition
Source: PLoS One. 2026 Mar 6;21(3):e0335625. doi: 10.1371/journal.pone.0335625 (PMC12965554; doi:10.1371/journal.pone.0335625)
Supplement: S9 Fig — (DOCX) [file pone.0335625.s014.docx]

**S9 Fig. Soil nitrate nitrogen content after three years of cropping.**


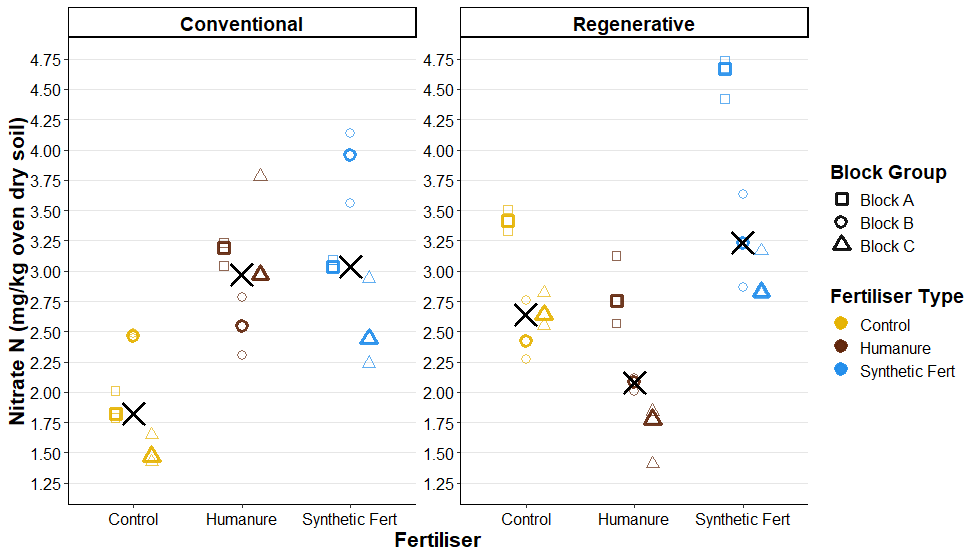

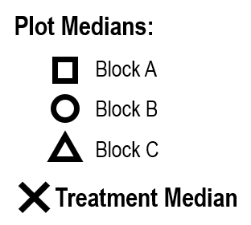


**S9 Fig. Soil nitrate nitrogen content after three years of cropping. Raw data points are displayed to show the degree of variation informing each plot median.**
