# Supplementary material for: The use of humanure for cereal production under conventional and regenerative farming models - findings from a three-year grassland-to-arable transition
Source: PLoS One. 2026 Mar 6;21(3):e0335625. doi: 10.1371/journal.pone.0335625 (PMC12965554; doi:10.1371/journal.pone.0335625)
Supplement: Attachment — Submitted filename: PONE-D-25-29073_Edited_Jenkins.pdf [file pone.0335625.s016.pdf]

PLOS ONE

The use of humanure under conventional and regenerative farming models - findings from a three year field trial

--Manuscript Draft--

**Manuscript Number:**	PONE-D-25-29073
**Article Type:**	Research Article
**Full Title:**	The use of humanure under conventional and regenerative farming models - findings from a three year field trial
**Short Title:**	Use of humanure under conventional and regenerative farming - three year field trial
**Corresponding Author:**	Katie Allen<br>University of Leeds<br>Leeds, UNITED KINGDOM OF GREAT BRITAIN AND NORTHERN IRELAND
**Keywords:**	humanure; ecological sanitation; EcoSan; field trial; plot trial; regenerative agriculture; regenerative farming; circular economy; circular sanitation; field research; soil health; human feces; human faeces; compost; composting; organic amendment; soil amendment
**Abstract:**	Humanure (composted human feces) offers a low-cost, simple treatment option for dry sanitation systems, and a promising organic amendment for crop production. However, no long-term studies have evaluated its impacts under different land management approaches. This study presents findings from a three-year field trial (2021–2024) in West Yorkshire, UK, assessing the effects of humanure on cereal crop yield and soil properties under conventional and regenerative farming practices. The experiment used a semi-randomised block design with two management regimes (conventional and regenerative) and three fertilisation treatments (control, synthetic fertiliser, and humanure), each replicated three times.<br>Crop results showed that humanure increased crop yields compared to the control, demonstrating a fertilisation effect, which was more pronounced under regenerative management. However, yields from humanure treatments were lower than those from synthetic fertilisers. Regenerative management produced higher yields than conventional across all fertiliser types.<br>Soil analyses revealed that fertiliser type had limited influence on physical soil properties, but regenerative practices significantly improved water-stable aggregates. Soil organic matter and total carbon were higher under humanure than other fertiliser regimes, and higher under regenerative management across all fertilisers. Humanure elevated phosphorus and potassium levels, but not outside of acceptable limits. Biological indicators, including worm counts, soil respiration, and fungal biomass, were slightly elevated under humanure treatments and consistently higher in regenerative systems. Due to small sample sizes, detailed statistical analysis was not appropriate, and instead trends are observed and discussed.<br>Overall, this study suggests that humanure is a promising soil amendment, especially when combined with regenerative land management. More investigation is warranted, with greater replication to enable statistical analysis, in order to further explore of the role humanure in realising a circular economy for sanitation and sustainable agriculture.
**Order of Authors:**	Katie Allen
	Effie Papargyropoulou
	Ruth Wade
	Barbara Evans
**Additional Information:**	
**Question**	**Response**
**Financial Disclosure**<br><br>Enter a financial disclosure statement that	The author(s) received no specific funding for this work.

describes the sources of funding for the work included in this submission. Review the submission guidelines for detailed requirements. View published research articles from *PLOS ONE* for specific examples.<br><br>This statement is required for submission and **will appear in the published article** if the submission is accepted. Please make sure it is accurate.<br><br>**Funded studies**<br>Enter a statement with the following details:<br>• Initials of the authors who received each award<br>• Grant numbers awarded to each author<br>• The full name of each funder<br>• URL of each funder website<br>• Did the sponsors or funders play any role in the study design, data collection and analysis, decision to publish, or preparation of the manuscript?<br><br>Did you receive funding for this work?	
**Competing Interests**<br><br>Use the instructions below to enter a competing interest statement for this submission. On behalf of all authors, disclose any competing interests that could be perceived to bias this work—acknowledging all financial support and any other relevant financial or non-financial competing interests.<br><br>This statement is required for submission and **will appear in the published article** if the submission is accepted. Please make sure it is accurate and that any funding sources listed in your Funding Information later in the submission form are also declared in your Financial Disclosure statement.<br><br>View published research articles from *PLOS ONE* for specific examples.	The authors have declared that no competing interests exist.

**NO authors have competing interests**<br><br>Enter: *The authors have declared that no competing interests exist.*<br><br>**Authors with competing interests**<br><br>Enter competing interest details beginning with this statement:<br><br>*I have read the journal's policy and the authors of this manuscript have the following competing interests: [insert competing interests here]*<br><br>* typeset	
**Ethics Statement**<br><br>Enter an ethics statement for this submission. This statement is required if the study involved:<br><br>• Human participants<br>• Human specimens or tissue<br>• Vertebrate animals or cephalopods<br>• Vertebrate embryos or tissues<br>• Field research<br><br>Write "N/A" if the submission does not require an ethics statement.<br><br>General guidance is provided below. Consult the submission guidelines for detailed instructions. **Make sure that all information entered here is included in the Methods section of the manuscript.**	N/A

Powered by Editorial Manager® and ProduXion Manager® from Aries Systems Corporation

**Format for specific study types**<br><br>**Human Subject Research (involving human participants and/or tissue)**<br>• Give the name of the institutional review board or ethics committee that approved the study<br>• Include the approval number and/or a statement indicating approval of this research<br>• Indicate the form of consent obtained (written/oral) or the reason that consent was not obtained (e.g. the data were analyzed anonymously)<br><br>**Animal Research (involving vertebrate animals, embryos or tissues)**<br>• Provide the name of the Institutional Animal Care and Use Committee (IACUC) or other relevant ethics board that reviewed the study protocol, and indicate whether they approved this research or granted a formal waiver of ethical approval<br>• Include an approval number if one was obtained<br>• If the study involved *non-human primates*, add *additional details* about animal welfare and steps taken to ameliorate suffering<br>• If anesthesia, euthanasia, or any kind of animal sacrifice is part of the study, include briefly which substances and/or methods were applied<br><br>**Field Research**<br><br>Include the following details if this study involves the collection of plant, animal, or other materials from a natural setting:<br>• Field permit number<br>• Name of the institution or relevant body that granted permission	
**Data Availability**<br><br>Authors are required to make all data underlying the findings described fully available, without restriction, and from the time of publication. PLOS allows rare exceptions to address legal and ethical concerns. See the PLOS Data Policy and FAQ for detailed information.	No - some restrictions will apply

A Data Availability Statement describing where the data can be found is required at submission. Your answers to this question constitute the Data Availability Statement and **will be published in the article**, if accepted.<br><br>**Important:** Stating 'data available on request from the author' is not sufficient. If your data are only available upon request, select 'No' for the first question and explain your exceptional situation in the text box.<br><br>Do the authors confirm that all data underlying the findings described in their manuscript are fully available without restriction?	
**Describe where the data may be found in full sentences. If you are copying our sample text, replace any instances of XXX with the appropriate details.**<br><br>• If the data are **held or will be held in a public repository**, include URLs, accession numbers or DOIs. If this information will only be available after acceptance, indicate this by ticking the box below. For example: *All XXX files are available from the XXX database (accession number(s) XXX, XXX.).*<br>• If the data are all contained **within the manuscript and/or Supporting Information files**, enter the following: *All relevant data are within the manuscript and its Supporting Information files.*<br>• If neither of these applies but you are able to provide **details of access elsewhere**, with or without limitations, please do so. For example:<br><br>*Data cannot be shared publicly because of [XXX]. Data are available from the XXX Institutional Data Access / Ethics Committee (contact via XXX) for researchers who meet the criteria for access to confidential data.*<br><br>*The data underlying the results presented in the study are available from (include the name of the third party*	Data available upon request.<br><br>I felt there was too much raw data to include in supporting information. I can put it onto some kind of repository if preferred.

and contact information or URL).<br>• This text is appropriate if the data are owned by a third party and authors do not have permission to share the data.<br><br>* typeset	
Additional data availability information:	Tick here if your circumstances are not covered by the questions above and you need the journal’s help to make your data available.

Manuscript Click here to access/download;Manuscript;Paper 3 for PLOS 28th May.docx

The use of humanure under conventional and regenerative farming models - findings from a three year field trial

Katie Allen[1]*, Effie Papargyropoulou[2], Ruth Wade[3], Barbara Evans[4].

1 School of Civil Engineering, University of Leeds

2 School of Earth and Environment, University of Leeds

3 School of Biology, University of Leeds

4 School of Civil Engineering, University of Leeds

*Corresponding author

E-mail: cnkma@leeds.ac.uk

Abstract

Humanure (composted human feces) offers a low-cost, simple treatment option for dry sanitation systems, and a promising organic amendment for crop production. However, no long-term studies have evaluated its impacts under different land management approaches. This study presents findings from a three-year field trial (2021–2024) in West Yorkshire, UK, assessing the effects of humanure on cereal crop yield and soil properties under conventional and regenerative farming practices. The experiment used a semi-randomised block design with two management regimes (conventional and regenerative) and three fertilisation treatments (control, synthetic fertiliser, and humanure), each replicated three times.

Crop results showed that humanure increased crop yields compared to the control, demonstrating a fertilisation effect, which was more pronounced under regenerative management. However, yields from humanure treatments were lower than those from synthetic fertilisers. Regenerative management produced higher yields than conventional across all fertiliser types.

Soil analyses revealed that fertiliser type had limited influence on physical soil properties, but regenerative practices significantly improved water-stable aggregates. Soil organic matter and total carbon were higher under humanure than other fertiliser regimes, and higher under regenerative management across all fertilisers. Humanure elevated phosphorus and potassium levels, but not outside of acceptable limits. Biological indicators, including worm counts, soil respiration, and fungal biomass, were slightly elevated under humanure treatments and consistently higher in regenerative systems. Due to small sample sizes, detailed statistical analysis was not appropriate, and instead trends are observed and discussed.

Overall, this study suggests that humanure is a promising soil amendment, especially when combined with regenerative land management. More investigation is warranted, with greater replication to enable statistical analysis, in order to further explore of the role humanure in realising a circular economy for sanitation and sustainable agriculture.

1. Introduction

It is estimated that global human fecal biomass production will exceed 1 billion tonnes by 2030 [1]. Yet currently only 57% of sanitation systems worldwide are safely managed [2], representing not only a public health crisis, but also a missed opportunity to capture and recycle the valuable nutrients and organic matter within fecal material.

At the same time, industrialised agriculture is a major driver of climate change, land degradation and diet-related health issues, and there is a growing recognition of the need to transition towards a more sustainable global food production model [3]. One aspect of this transition is reducing reliance

on synthetic fertilisers, which contribute significantly to greenhouse gas emissions [4], and create widespread economic and social vulnerabilities due to dependence on volatile global supply chains [5, 6]. This may be achieved by directly substituting synthetic fertilisers with a more sustainable nutrient source, or by adopting farming practices which do not require such high chemical inputs.

Together, these drivers present a compelling case for exploring the use of human fecal material in agricultural production, due to its abundance, locality, affordability and nutrient content. It offers a promising option for both sanitation management and as part of a more sustainable food system, and closes the loop on nutrients between sanitation and food production.

Previous research on the agricultural use of human fecal material is concentrated on wastewater and biosolids; the liquid and semi-solid outputs of sewer-based wastewater treatment plants. Comparatively little attention is given to alternative toilet systems, which contain the material in situ and require periodic emptying [7]. Despite representing the most common form of sanitation service globally [2], these systems have been largely overlooked in the context of agricultural reuse.

Dry toilets which do not use flushwater are one such system, and require different management to sewage. One promising treatment option which is suitable for agricultural reuse is composting, due to its simplicity, low cost, and broad applicability [8]. In contrast to extractive technologies (e.g. phosphorus extraction [9]), composting utilises the entirety of the toilet material, aligning it with zero waste and circular economy principles [10, 11]. Composted human feces are henceforth referred to as 'humanure', a term popularised by Jenkins [12].

Previous studies on the land application of humanure have reported improved crop growth relative to unfertilised controls, as well as increases in soil organic matter (SOM), nitrogen, and phosphorus levels—indicating a promising fertilisation effect [13-15]. However, a recent systematic review of faecal amendments in agricultural trials [7] highlighted a critical lack of multi-year field studies and insufficient reporting on key soil health indicators, particularly those related to physical and

biological properties. This limits our understanding of the longer-term and systemic impacts of humanure on soil function.

Moreover, existing research on feces-based amendments has typically been conducted without situating trials within clearly defined or deliberately modelled farming systems. As a result, we lack understanding of how the performance of such amendments differs under varying land management approaches. With growing recognition of the need to transition toward more sustainable agricultural practices, it is important that research into alternative fertilisers—such as humanure—considers their role within the full context of different farming systems, as such amendments are likely to behave differently under different management practices. Understanding these interactions is critical for assessing the real-world viability of humanure and identifying contexts in which it can most effectively contribute to soil health and crop production.

The present study was designed to examine the effects of humanure on crop growth and soil health over three years in a field plot experiment, following the trial design recommendations outlined in the aforementioned systematic review [7]. It compares the performance of humanure to synthetic fertiliser and to an unamended control under two different land management strategies; a 'conventional' system and a 'regenerative' system.

Terms used to describe agricultural systems are often oversimplified or politicised, obscuring the immense diversity of farming practices [16]. As recommended by Newton et al. (2020) [17] a detailed list of the specific "conventional" and "regenerative" practices used within this study is provided in the supplementary materials, and these terms are used as a tool to simplify the discussion. The authors acknowledge the complexity of farming systems and understand that the results presented here do not represent all systems globally. Rather, it is hoped that this exploratory study will highlight interesting trends and inform future research in the field.

1.1 Conventional Management Experiment

What is considered ‘conventional’ varies globally, making cross-study comparison and broad generalisation challenging. Typically, conventional agriculture refers to large-scale, mechanised farming operations with minimal crop diversity (e.g. monocultures) and a high reliance on external inputs of crop nutrients and other agrichemicals. This study models a typical UK cereal production system, incorporating mechanical tillage and pesticide use. More details are presented in the Methods section.

The primary objectives of this system within the study are to offer comparison against typical ‘best practice’ for UK cereal production, and to evaluate the direct substitution of synthetic fertiliser with humanure within such a system.

Synthetic fertilisers are designed for use in conventional, intensive farming systems, where they supply highly soluble forms of nitrogen, phosphorus, and potassium (NPK) that are immediately available for plant uptake. In such systems, soil biological processes that typically support nutrient cycling are often disrupted—due to factors like intensive tillage and chemical pest control—which impairs the soil's natural ability to supply endogenous nutrients to plants [18-21]). Synthetic fertilisers effectively bypass the need for a healthy, functioning soil ecosystem by directly delivering nutrients, making them an attractive solution in degraded or biologically simplified systems.

In contrast, humanure contains large quantities of these elements in organic forms, which require mineralisation by soil microorganisms before they can become available to plants, resulting in a slower release of plant-available nutrients [22, 23].

Therefore, it is theorised that direct input substitution of synthetic fertiliser for humanure would see an initial reduction in crop yield, but that this yield gap may close over multiple seasons as the nutrients become available. Additionally, it is theorised that, due to its organic matter content,

humanure will offer benefits for soil health which are not observed under synthetic fertilisation [13, 15, 24-29].

The review by Allen et al. (2023) found only four high quality studies to date which had directly compared the effects of humanure and synthetic fertiliser on crop growth [15, 30-32], all of which took place over a single cropping cycle of a few months, highlighting the need for more long-term research.

1.2 Regenerative Management Experiment

'Regenerative' agriculture also encompasses a wide range of practices [33]. Broadly, it aims to restore soil and ecosystem health through methods including minimising soil disturbance, maintaining living roots year-round, and increasing plant diversity. In this study, the regenerative system includes minimal tillage, no agrichemical inputs, cover cropping, and a ley year without a market crop (full details in the Methods section).

The objective of this system within the study is to assess how humanure might contribute to soil regeneration, and how it may offer greater fertility benefits within a regeneratively managed system as opposed to a conventional one.

Since the organically-bound nutrients contained within organic materials require mineralisation to become plant-available [22], it is theorised that the fertilising capability of humanure will depend not only on its nutrient content, but also on the biological activity and overall health of the surrounding soil system. Given this, humanure is expected to deliver comparatively greater crop fertilisation effects under systems which focus on regeneration of soil ecosystems and promotion of biological soil health.

At the time of publication the authors were not aware of any previous studies investigating the use of humanure under regenerative or other sustainable agricultural practices.

2. Materials and Methods

2.1 Experiment Overview

A plot trial was conducted over three years with three crop growth cycles, from June 2021 to September 2024.

It followed a 3x2 factorial design, where six treatment/management combinations were applied across the study site, each replicated three times. The three fertiliser treatments were a control (no fertiliser), humanure and synthetic fertiliser, which were applied based on a matched total nitrogen loading rate. The two land management types were conventional and regenerative practices.

This approach allowed for the assessment of both individual effects of fertiliser type and land management, as well as potential interactions between these factors.

Multiple parameters of crop growth and soil health were measured following the third cropping cycle. This study reports the crop and soil results from the end of the third cropping cycle, in September 2024.

2.2 Site Details

The plots were located on the University of Leeds research farm, located in Tadcaster, West Yorkshire (53°52'00.0"N 1°19'51.0"W). The study site covered an area of 0.1ha which had previously been used for grass growing trials. The soil is a clay loam texture.

The plots are located within a Nitrate Vulnerable Zone, which placed restrictions on the timing and quantity of Nitrogen applications from different sources.

2.3 Plot Layout

The research area measured 48 x 25m and was divided into 24 plots of 1.5m width and 12m length (Figure 1). The layout was semi-randomised block design, comprising three blocks (A, B and C). Each

block contained one regenerative management row and one conventional management row. This design enabled access to the entire row for easier use of machinery. Each row contained one of each fertiliser treatment and a discard plot, with positions within the row randomised. Fertilisation was performed by hand.

Figure 1 – Experimental plot layout and plot codes. Vertical rows alternate between conventional (pink) and regenerative (green) management, separated by a 2m gap. Each row included one of each of the treatments in a randomised position; control (yellow), humanure (brown), synthetic fertiliser (blue) and a discard plot (grey).

2.4 Baseline Soil Assessment

Prior to the start of the experiment, the research area was ploughed and treated with herbicide (Motif, 5L/ha), before the entire area was power harrowed twice in September. This terminated weeds and prepped a uniform bed to begin the trial.

Baseline soil testing was conducted in September 2021, the day before the first crops were planted. The sampling protocol was adapted from the Cornell Framework [34]. Ten samples were taken from around the site in two W shapes and mixed to produce one composite sample. The sampling depth was 15cm.

2.5 Land and Crop Management

The trial compared two different management practices; termed “conventional” and “regenerative”.

Table 1 highlights the key management differences between the conventional and regenerative plots. The conventional management approach was modelled on the AHDB growth guides for wheat and barley [35, 36]. A more detailed list of plot management activities is included in the Supporting Information (S1).

Table 1 – Overview of key management activities undertaken on the conventional and regenerative plots. Full details are given in the Supporting Information (S1).

	Conventional	Regenerative
Ground preparation	Deep plough (8 inches) each year to terminate weeds and bury crop residue.<br>Surface power harrowed each year to prepare seedbed for planting.	Surface power harrowed prior to Year 3 sowing to penetrate thick crop residue.
Sowing	Direct drill and rolled	Direct drill and rolled
Crops	Year 1 – Winter wheat (*Syngenta Gleam*)<br>Year 2 - Spring barley (*LG Diablo*)<br>Year 3 - Spring Barley (*Laureate*)	Year 1 - Winter wheat (*Syngenta Gleam*) + red clover (*Avisto*) understory<br>Year 2 - Living mulch of red clover (*Avisto*) and fodder radish.<br>Year 3 - Spring Barley (*Laureate*)
Chemical Sprays	Pre-emergence herbicide, and fungicide application as recommended in the AHDB growth guides. Spot use of glyphosate for weed control as required.	None
Winter cover	Fallow	Cover crop of red clover, mowed off prior to Y3 sow.

The crops and varieties selected were typical for a UK cropping rotation, consisting of winter wheat, followed by two years of spring barley.

2.6 Fertiliser Applications

The trial compared the impacts of 3 different sources of crop nutrition. These are:

- Control (Con) - No fertiliser application
- Synthetic fertiliser (SF) – Commercial solid prilled NPK fertiliser ('Origin'), 20-10-10 compound blended mix. These contain (by weight) 20% total N, 10% phosphate (P_2O_5) and 10% potash (K_2O).
- Humanure – Composted human feces and sawdust aged for at least 2 years.

The humanure and SF were surface applied by hand during periods of dry weather.

The SF was applied at the recommended nitrogen loading rate and timings, from the AHDB crop growth guides [35, 36] and consultation with the site trial manager.

Each humanure batch was sourced from different locations around the UK, meaning that its composition differed each year. A summary of the batch collection and management, as well as a full compositional analysis, is given in the Supporting Information (S2).

The nitrogen composition of the humanure was used to calculate the quantity of humanure to be applied to the plots each year, in order to closely match the total nitrogen (N_{tot}) loading rate between the humanure and synthetic fertilisers (Table 2). For practicality, this was done by calculating the number of kilograms of humanure required to be added to each plot, based on the N_{tot} concentration and moisture content.

The uncertainty around the mean applied values is expressed in Table 2 as upper and lower bounds, shown in brackets. This uncertainty comes from the variance in N_{tot} content, moisture content, and error associated with weighing.

In addition to N_{tot}, Table 2 also shows the calculated doses of total nitrogen (N_{tot}), inorganic nitrogen (N_{in}), organic matter (OM), carbon (C_{tot}), phosphorus and potassium which were applied to each plot

each year. For synthetic fertiliser, the quantities of total elemental phosphorus (P_{tot}) and total potassium (K_{tot}) were calculated from the dose of NPK 20:10:10 applied, which is 10% phosphate (P_2O_5) and 10% potash (K_2O) by mass. For humanure it was not possible to calculate P_{tot} and $K_{tot.}$ Instead available P (P_{av}) and exchangeable potassium cations (K+) were measured in the labs. The same lab methods used for soil analysis given in the Methods section were also used for the humanure batch analysis.

Table 2 – Estimated quantity of Nitrogen, and Organic Matter, Phosphorus and Potassium added to each of the plots each year. Numbers in brackets indicate the maximum and minimum possible values based on the error.

		Year 1*		Year 2		Year 3	
		Humanure 1	SF	Humanure 2	SF 2	Humanure 3	SF 3
Recommended dose and timing N_{tot} (kgN/ha)		*Early Spring - 50*<br>*Mid Spring - 100*<br>*Late Spring - 100*		*Late Spring – 140*		*Mid Spring - 65*<br>*Late Spring - 65*	
**N_{tot}**	**kg/ha**	**232 [181, 339]**	**250**	**156 [139, 169]**	**140**	**198 [180, 268]**	**130**
N_{In}	kg/ha	48.23 [16.65, 78.56]	250	32.96 [26.54, 40.16]	140	20.19 [16.15, 57.49]	130
OM	t/ha	6.98 [5.45, 9.37]	-	6.19 [5.62, 6.80]	-	6.43 [5.73, 8.69]	-
C_{tot}	t/ha	3.15 [2.69, 4.07]	-	2.45 [2.24, 2.66]	-	3.05 [2.77, 4.11]	-
P_{av}	kg/ha	1.95 [1.61, 2.65]	-	16.78 [13.24, 20.77]	-	10.88 [5.94, 15.78]	-
P_{tot}	kg/ha	-	54.55	-	30.55		28.37

K+	kg/ha	36.21 [7.12, 82.41]	-	130.79 [110.68, 153.09]	-	no data	-
K_{tot}	kg/ha	-	103.75	-	58.1	-	53.95

**An additional 50kg/ha of N_{in} and 10.9kg/ha of $P_{available}$ was erroneously applied to all plots in the first year, including the control plots in the form of 20:10:10 NPK Synthetic Fertiliser (262g).*

2.7 Sampling and Laboratory Tests

2.7.1 Soil Sampling and Laboratory Work

Soil samples were taken at the end of Year 3. Five samples were taken from each plot using the same Cornell method as used for baseline soil analysis [34], to a depth of 15cm. These were combined to create one composite sample for each plot. Composite samples were sieved to 8mm to remove large stones and roots, then half of the material was stored fresh at 4°C, whilst the other was air dried.

Bulk density samples were collected using a soil corer with a cutting shoe. The internal cylindrical rings were 5cm diameter and 5cm height (volume 98.17cm^3). These were collected at a depth of 5-10cm, by removing the first 5cm of topsoil before sampling.

Lab analyses were conducted to determine key physical, chemical and biological properties of the soil. Table 3 summarises the methods used.

Table 3 – Summary of measured soil parameters and laboratory methods used.

**Parameter**	**Analysis Method**	**References**
Bulk density	Oven dried at 105°C for 12 hours then weighed.	[37]
Water Stable Aggregates	Air dry soil sieved to 2mm, placed onto a 1mm sieve and submerged in water for 5 mins, then removed and submerged again 5 times to dislodge slaked soil, before	[38]

	removal, drying and weighing to determine proportion retained on the sieve. Indicates the stable fraction of particles sized between 1mm and 2mm.	
pH	Analysed using Oakton® pH 700 Benchtop pH Meter -	[39]
Moisture	Oven dried at 105°C for 12hrs, then weighed.	
Soil Organic Matter (SOM)	Loss on ignition.<br>Heated to 550°C for 12 hours, then weighed.	[40]
Total carbon (C_{tot}) and total nitrogen (N_{tot})	Air-dried soil ground on a Resch mixer mill MM400.<br>Analysed using an Elementar analyser vario MICRO cube.	[41, 42]
Inorganic nitrogen (N_{In}), nitrate (NO_3_N) and ammonium (NH_4_N)	Potssium chloride extraction.<br>Analysed using a Skalar SAN++ continuous flow auto-analyser and spectrophotometric detection.	[43, 44, 45]
Available phosphorus (P_{av})	Olsen's P method.<br>Sodium bicarbonate extraction.<br>Analysed using a Skalar SAN++ continuous flow auto-analyser and spectrophotometric detection	[39, 46]
Exchangeable potassium (K+)	Ammonium chloride extraction.<br>Analysed using ICP-OES Thermo Fisher ICAP 7600.	[43, 47, 48]

Worm Count (field measure)	Three 15cm^3 holes were dug at a random positions within each 1/3 of each plot. For each, the material was broken apart by hand and all worms were enumerated.	
Microbial respiration	Fresh soil incubated with sodium hydroxide for seven days, followed by titration with hydrochloric acid.	[39]
Fungal biomass	Measuring the size of fungi observed by direct microscopy of a known dilution of soil.<br>25 fields of view are enumerated per sample droplet which is used to estimate the total fungal biomass within the droplet.	Method developed and taught on the microscopy course offered by the Soil Ecology Lab, Hampshire, UK.

2.7.2 Plant Sampling and Laboratory Work

Establishment was measured in May of 2024, the final year of the experiment, using a 0.25m^2 quadrat. Three locations were sampled per plot; the plot was divided into three equal sections along its length, and a sample was taken from a random location within each of these sections. The outermost rows were avoided to minimise edge effects. The number of plants within the quadrat was counted.

One week before harvest, in August 2024, eight plants were harvested by hand from each plot in order to measure growth metrics. No plants were taken from the endmost one metre, nor from the outermost rows to avoid edge effects, but otherwise the position in the stand was randomised. These samples were used to determine plant height, tiller count and number of grains-per-head, before they were oven dried at 40°C to constant weight in order to measure dry biomass.

A visual assessment of disease and damage to the grain heads was also performed, and each plot batch was qualitatively classified as having none (0% of heads affected), mild (<20% affected),

moderate (20-40% affected) or severe (>40% affected) disease/damage losses (Supporting Information S3). This is because head losses to damage and disease are a contributing factor to lower overall yields.

Total yield measures for the plots were determined at harvest in August 2024, when grain moisture content was 17%. A sample of each yield batch was then dried for 24 hours to a moisture content of 12-14%, which was used for the thousand grain measurement.

2.8 Statistical Analysis

Data were recorded and cleaned in Microsoft Excel, which was also used to determine median and range values. Graphs were created in R Studio version 4.3.2.

Due to the small sample size of just three treatment replicates, the median was used as the most appropriate measure of central tendency, and only descriptive statistics are reported. Differences and trends are described and offer interesting avenues for further research.

For each plot, multiple raw values were measured to determine the plot median (M_{plot}). These three plot medians were then used to inform the treatment median ($M_{treatment}$). In tables, these treatment medians are reported as $M_{treatment}$ [Lowest M_{plot} , Highest M_{plot}] allowing all three M_{plot} values to be easily shown.

The scatter plots show all of the raw data points which inform M_{plot}, disaggregated by fertiliser treatment and management type. This shows the entire spread of the dataset, the raw sample size for each parameter, and allows for easy visualisation of any trends, or any location effects of the three trial blocks.

The interaction plots are plotted using the $M_{treatment}$ values, and show overall trends between the fertiliser types and between management types, as well as any possible interactions between the two factors.

3. Results

3.1 Crop Results

3.1.1 Yield

The spring barley was harvested and weighed at 17% moisture content.

Under conventional management, synthetic fertiliser produced the highest yields (yield $M_{treatment}$ = 1.39t/ha [1.00, 2.49]), followed by humanure (yield $M_{treatment}$ = 1.01t/ha [0.95, 1.47]) then the control (yield $M_{treatment}$ = 0.67t/ha [0.58, 0.88]), with some overlap (Fig 2).

Under regenerative management, synthetic fertiliser produced the highest yields (yield $M_{treatment}$ = 4.15t/ha [1.42, 4.94]), followed by humanure (yield $M_{treatment}$ = 2.94t/ha [2.20, 3.10]) then the control (yield $M_{treatment}$ = 0.82t/ha [0.64, 1.24]), however the graph reveals the large variance for SF, and subsequent overlap.

The interaction plot reveals that regenerative management had a positive impact on yield as compared with conventional management, resulting in higher yields under regenerative management for all fertiliser treatments. This effect particularly pronounced for the humanure and SF plots, indicating a positive interaction effect. This means that under conventional management, the addition of fertiliser only contributed towards a small yield increase, whereas under regenerative management a humanure and SF fertilisation produced an even larger yield increase than would be expected from fertilisation alone.

Fig 2 – Spring barley yield at harvest of the third cropping season, under conventional and regenerative management; 17% moisture content.

3.1.2 Other Crop Growth Measures

Other measures of crop growth provide greater insight into the differences observed in overall yields (Table 4). Graphical representation of these results are included in the Supporting Information (S4).

Table 4 – Treatment medians for six crop growth parameters for the spring barley harvested in August 2024. Numbers in brackets indicate the range of the plot medians. N = the number of raw data points which informed each of the plot medians.

		Conventional			Regenerative		
**Parameter**	**Unit**	**Control**	**Humanure**	**SF**	**Control**	**Humanure**	**SF**
Establishment (n = 3)	Plants per 0.25m^2	57 [37,58]	67 [45, 70]	57 [44, 58]	49 [40, 53]	48 [42, 50]	37 [21, 41]
Tillers (n = 8 plants)	Tillers per plant	3 [2.5, 4]	5.5 [5, 6]	6 [5, 7]	6 [4, 6]	5 [3, 5.5]	4.5 [4, 7]
Height (n = 8 plants)	cm	24.5 [22, 25]	35.5 [25.5, 41.5]	42 [37.5, 51.5]	35 [29.5, 46.5]	45.5 [38, 55.5]	62 [43.5, 67]
Grain Number (n = 8 plants)	Grains per head	6 [5, 7.5]	10 [7.5, 15.5]	16 [10.5, 19.5]	9.5 [9, 11]	15 [11, 24]	24.5 [16, 25]
1000 grain mass (n = 1)	g	44.0 [43.4, 46.8]	47.0 [43.4, 50.1]	46.8 [36.1, 50.8]	38.1 [33.7, 51.7]	39.3 [37.1, 43.4]	47.8 [40.3, 51.9]
Dry Head Biomass (n = 1)	g per head	0.19 [0.13, 0.26]	0.21 [0.18, 0.32]	0.56 [0.43, 1.19]	0.35 [0.30, 0.45]	0.63 [0.40, 1.41]	1.46 [0.50, 1.65]
Head damage / disease (n = 1)	Qualitative assessment*	mild+	moderate+	mild	mild+	none+	none+

**The modal disease classification of the three plots is reported. Where one plot was worse than the other two, a '+' is added to the classification.*

Under conventional management, the higher SF yield is likely attributable to higher tillering, higher number of grains per head and lower damage losses than humanure and the control. Humanure had the best establishment (Establishment $M_{treatment}$ = 67 plants per 0.25m^2 [45, 70]) but lower grain

number, which lead to a lower head biomass. The control plot performed the lowest across all growth parameters, which correlated with the low overall yield.

Under regenerative management, the higher SF yield is likely attributable to the high number of grains per head (Grain Number $M_{treatment}$ = 24.5 grains per head [16, 25]).

The fact that yields were higher under regenerative management than conventional management for all three fertilisation treatments is interesting, considering that the regenerative plots saw lower establishment and lower 1000 grain mass (except for SF treatment), and similar tillering. Grain number was consistently higher in the regenerative plots, and thus appears to be the strongest predictor of yield. This theory is strengthened by the fact that within each management group, the grain number was well correlated with the yield performance for each of the three fertilisers. Additionally, disease and damage was consistently lower under the regenerative management.

3.2 Soil

Sixteen parameters of soil health were assessed after three years of cropping (Table 5). Additional graphical representations of some parameters which are not included in the manuscript are available in the Supporting Information (S5).

Table 5 – Treatment medians for 16 soil parameters, prior to the start of the experiment (baseline) and after three years of cropping. Numbers in brackets indicate the range of the plot medians. N = the number of field/lab replicate analyses which informed each of the plot medians.

			**Conventional**			**Regenerative**		
Variable	Unit	Baseline Soil‡	Control	Humanure	SF	Control	Humanure	SF
**Bulk Density** (n = 2)	g cm^{-3} oven dry soil	1.54 [1.48, 1.56]	1.51 [1.47, 1.52]	1.52 [1.50, 1.55]	1.54 [1.47, 1.55]	1.53 [1.42, 1.53]	1.51 [1.44, 1.58]	1.48 [1.45, 1.55]

**Water Stable Aggregates** (n = 3)	%	-	61.45 [53.34, 63.04]	56.11 [53.54, 58.05]	60.46 [51.93, 67.41]	73.5 [72.13, 76.66]	79.25 [76.5, 83.33]	82.33 [69.27, 82.36]
**pH** (n = 2)		7.49 [6.89, 7.55]	7.05 [6.96, 7.15]	7.29 [7.27, 7.44]	7.16 [7.15, 7.21]	7.54 [7.56, 6.90]	7.27 [7.25, 7.27]	7.39 [7.31, 7.48]
**Moisture** (n = 3)	% field moist soil	14.57 [14.14, 14.97]	18.37 [18.57, 17.99]	18.59 [17.66, 20.25]	18.44 [17.81, 18.47]	18.41 [18.05, 19.03]	19.48 [19.38, 19.52]	18.43 [18.19, 18.62]
**Organic Matter** (n = 3)	% oven dry soil	4.70 [4.63, 4.85]	4.56 [4.44, 4.61]	4.87 [4.75, 5.54]	4.54 [4.38, 4.63]	4.82 [4.74, 5.01]	5.28 [5.08, 5.33]	4.73 [4.68, 4.97]
**Total C** (n = 2)	% oven dry soil	1.57 [1.55, 1.65]	1.46 [1.45, 1.51]	1.61 [1.55, 1.69]	1.41 [1.41, 1.54]	1.68 [1.59, 1.79]	1.75 [1.68, 2.06]	1.50 [1.49, 1.58]
**Total N** (n = 2)	% of oven dry soil	0.15 [0.14, 0.15]	0.15 [0.14, 0.20]	0.19 [0.19, 0.23]	0.21 [0.20, 0.22]	0.22 [0.11, 0.23]	0.20 [0.20, 0.28]	0.17 [0.14, 0.22]
**Inorganic N** (n = 3)	mg/kg oven dry soil	15.97 [14.97, 16.49]	2.54 [2.10, 3.10]	3.91 [3.28, 3.94]	3.68 [3.12, 4.44]	3.62 [3.21, 4.18]	2.80 [2.38, 4.35]	3.96 [3.70, 5.44]
**NH_4_N** (n =3)	mg/kg oven dry soil	1.42 [1.34, 1.57]	0.63 [0.55, 0.67]	0.83 [0.72, 0.88]	0.61 [0.58, 0.70]	0.75 [0.69, 0.86]	0.76 [0.63, 1.52]	0.77 [0.66, 0.80]
**NO_3_N** (n = 3)	mg/kg oven dry soil	14.34 [13.49, 14.94]	1.82 [1.47, 2.46]	2.97 [2.54, 3.19]	3.04 [2.44, 3.96]	2.64 [2.42, 3.42]	2.08 [1.78, 2.75]	3.23 [2.83, 4.67]
**C:N** (n = 3)		11.18 [10.72, 11.29]	10.18 [7.41, 10.77]	8.73 [6.65, 9.07]	6.66 [6.43, 7.95]	8.30 [6.81, 15.40]	8.39 [6.32, 10.37]	8.81 [7.36, 10.82]

**Available P** (n = 3)	mg/kg oven dry soil	6.23 [6.10, 7.46]	6.53 [5.24, 7.13]	17.97 [12.95, 25.96]	8.08 [7.77, 8.70]	4.41 [4.37, 4.73]	16.72 [8.63, 23.80]	5.69 [4.77, 6.18]
**K+** (n = 3)	mg/kg oven dry soil	-	108.77 [104.44, 114.78]	149.79 [149.67, 201.61]	117.01 [110.60, 151.70]	123.25 [122.41, 123.78]	174.65 [109.51, 175.75]	144.03 [113.47, 144.47]
**Respiration** (n = 3)	g CO2 kg-1 dry soil y-1	0.32 [0.30, 0.35]	1.05 [0.97, 1.20]	1.66 [1.66, 1.75]	1.23 [1.09, 1.42]	1.39 [1.22, 1.49]	1.73 [1.46, 1.95]	1.57 [1.41, 1.88]
**Worms** (n = 3)	worms per $15cm^3$	-	11 [9, 12]	13 [12, 13]	11 [9, 13]	17 [16, 18]	23 [22, 23]	18 [17,18]
**Fungi** (n = 3)	ug ml^{-1} air dry soil	-	2.07 [0.00, 15.53]	5.87 [4.10, 6.21]	1.90 [0.52, 2.15]	7.07 [5.52, 15.68]	16.18 [12.66, 20.70]	9.05 [5.87, 13.11]

‡ n = 3; 5 grab samples compiled into one mixed bulk sample, then measured in triplicate. Reported as median [range low, range high].

3.2.1 Bulk Density

Bulk density did not change measurably throughout the experiment duration, with all treatment median values falling within the recorded range of the baseline soil for both management types (Supporting Information S5).

3.2.2 Water Stable Aggregates

Under conventional management, humanure exhibited a slightly lower median WSA than the control and SF treatments (humanure WSA $M_{treatment}$ = 56.11% [53.54, 58.05]), but there was a high degree of overlap between the different treatment plots, so this difference is not considered meaningful (Fig 3). All nine conventional M_{plot} values fell within 51.9% and 67.41%.

Fig 3 – Water stable aggregates after three years of cropping. Percentage of air-dried particles retained on a 1mm sieve after submersion and agitation.

Under regenerative management, wet aggregate stability was again similar between the three fertiliser treatments, with a high degree of overlap between the different treatment plots. Synthetic fertiliser reported the highest median value in plot R04_SF (Block A), but also the greatest range (SF WSA $M_{treatment}$ = 82.33% [69.27, 82.36]). All nine regenerative M_{plot} values fell within 69.27% and 83.33%.

This indicates that fertiliser treatment did not have a meaningful impact on WSA, with a high degree of overlap and similar variance between each fertiliser treatment within each management group. However, there is a clear impact of management on water stable aggregates, with the regenerative practices resulting in higher WSA across all three fertiliser treatments.

The interaction plot reveals this effect clearly, with a large difference in $M_{treatment}$ values between conventional and regenerative management. It also offers a slight indication that these increases under regenerative management are less pronounced for the control treatment as opposed to the humanure and SF treatments, but given the variance of the raw data and the small sample sizes this finding is not considered meaningful.

3.2.3 pH

No clear directional effect of management nor fertiliser treatment was observed for pH. All M_{plot} values fell within the recorded range of the baseline soil (6.98 – 7.55), except for plot R08_Control (Block B) where M_{plot} = 7.56 (Fig 4).

Fig 4 – Soil pH after three years of cropping. The red line shows the baseline soil median value, and the grey lines show the baseline soil value range.

There was no clearly observed trend between treatments within each management type, nor between the management types. Data clustering was generally good, except for plot R03_Control

(Block A) which was much lower than the rest of Block A and the rest of the Regenerative-Control plots.

3.2.4 Moisture

Moisture content reflects a snapshot of the field conditions on one given day, and is primarily used in lab calculations to convert between wet and dry masses. As such, it was not compared to any baseline value, however internal experimental comparisons are still of interest.

Under conventional management, humanure displayed the widest range of moisture content values across the field, from driest in Block A (northwest) to wettest in Block C (southeast) (Fig 5). The control treatment and SF treatments were more clustered, and so there so not appear to be clear location-linked effects. Given this wide range, it is difficult to comment on the relative effect of humanure on moisture content under conventional management.

Fig 5 – Soil moisture content.

Under regenerative management a clearer difference can be observed between the fertiliser treatments due to smaller variance, with humanure plots displaying a higher median moisture content than SF and control treatments (humanure Moisture $M_{treatment}$ = 19.48% [19.38, 19.52]).

No clear difference is observed between management types.

3.2.5 Organic Matter and Carbon

Under conventional management, humanure resulted in the highest SOM (humanure SOM $M_{treatment}$ = 4.87% [4.75, 5.54]), with the median exceeding the upper range of the baseline soil (baseline SOM $M_{Treatment}$ = 4.70% [4.63, 4.85]. Both the control and SF treatments showed very similar SOM values, which were below the baseline SOM range, indicating a small decline (Fig 6).

Fig 6 – Soil organic matter content after three years of cropping. The red line shows the baseline soil median value, and the grey lines show the baseline soil value range.

Under regenerative management, humanure clearly showed a higher SOM value than the other two fertiliser treatments (humanure SOM $M_{treatment}$ = 5.28% [5.08, 5.33]), and well exceeded the baseline SOM value range, indicating a meaningful increase. The control performed slightly better than the SF treatment, and both were within the upper range of the baseline soil.

The interaction plot indicates a clear positive effect of regenerative management on SOM, with all treatments performing better under this management type, and a clear positive effect of humanure on SOM under both management types. No clear interaction effect is observed between the two, as the interaction lines are broadly parallel.

Total elemental carbon (C_{tot}) followed a similar trend to SOM, with humanure resulting in the highest C_{tot} under both management types, and with regenerative management resulting in higher C_{tot} than conventional management across all fertiliser treatments. One key difference was the much larger increase in C_{tot} between conventional and regenerative management for the control treatment, as compared to the degree of increase in the humanure and SF treatment. Another was the large variation in the regenerative humanure samples.

3.2.6 Nitrogen

Under conventional management, both humanure and SF treatment raised the Total Elemental Nitrogen (N_T) in soil compared to the baseline value as well as the control plots (Fig 7). Under regenerative management no clear trend was observed between the fertiliser treatments, due to high variation and overlap. The interaction plot reveals no clear directional effect of management type nor fertiliser type on N_T, suggesting that they are not significant predictors of N_T.

Fig 7 – Soil total elemental nitrogen content after three years of cropping. The red line shows the baseline soil median value, and the grey lines show the baseline soil value range.

Inorganic N (N_{In}) only comprised around 0.1% of the total elemental N (N_T) in the soils. All values were significantly below the baseline soil values (baseline N_{In} $M_{treatment}$ = 15.97mg/kg [14.97, 16.49]), and ranged from 2.10-5.44mg/kg (Fig 8).

Fig 8 – Soil inorganic nitrogen content after three years of cropping. The baseline values were significantly higher than the recorded values, and so are not shown on the graph.

Similarly to N_T, under conventional management N_{In} was elevated in the humanure and SF fertiliser treatments as compared with the control treatment. This trend was not observed under regenerative management, where all three treatments showed high variation and large overlap. The interaction plot reveals no clear directional effect of management type nor fertiliser type on N_{In}, suggesting that they are not significant predictors of N_{In}.

Nitrate N (NO_3^-_N) comprised a large proportion of N_{In} (typically upwards of 75%), and subsequently followed a very similar distribution (Supporting Information S5). Like N_{In}, all observed NO_3^-_N M_{plot} values were significantly lower than the baseline soil (baseline NO_3^-_N $M_{treatment}$ = 14.34mg/kg [13.49, 14.94]), ranging from 1.47-4.67mg/kg. No clear directional effect of management type nor fertiliser type is observed.

Ammonical N (NH_4^+_N) comprised a smaller proportion of N_{In} than nitrate. All treatments across both management types resulted in NH_4_N concentrations below the baseline soil range (baseline NH_4^+_N $M_{treatment}$ = 1.42mg/kg [1.34, 1.57]) (Supporting Information S5). No meaningful differences were observed between groups, with all $M_{treatment}$ values falling within 0.61 – 0.83 mg/kg, and no clear effect of management type nor fertiliser treatment was observed.

3.2.7 Available Phosphorus

Under both conventional and regenerative management, humanure greatly elevated the available phosphorus (P_{av}) compared with the baseline soil (conventional humanure P_{av} $M_{treatment}$ = 17.97mg/kg [12.95, 25.96]; regenerative humanure P_{av} M = 16.72mg/kg [8.63, 23.80]; baseline

humanure P_{av} $M_{treatment}$ = 6.23mg/kg [6.10, 7.46]) (Fig 9). Both management types also showed a high degree of variation between the three replicate humanure plots, whereas the control and SF plot results were better clustered. Both the control and SF treatments correlated closely with the baseline values.

Fig 9 – Soil available phosphorus after three years of cropping. The red line shows the baseline soil median value, and the grey lines show the baseline soil value range. The green lines show the boundaries for Index 2, the recommended range for arable crops.

This shows a clear, direct effect of humanure on raising P_{av}, and indicates that the fertiliser type was a much more significant predictor of P than the management type. However, there still appears an effect of management type on P_{av}, with $M_{treatment}$ values consistently slightly lower under regenerative management than conventional management across all treatments, as clearly shown on the interaction plot. This trend of lower P_{av} under regenerative management, as compared with conventional management, was seen across all Block paired comparisons, except for Block B_Humanure (brown circles) which showed the opposite relationship.

Humanure treated raised P_{av} to within the Index 2 range, the recommended range for arable crops [49].

3.2.8 Potassium

Under conventional management, humanure elevated potassium cations (K+), as compared with the control and SF treatments (humanure K+ $M_{treatment}$ = 149.79mg/kg [149.67, 201.61]) (Fig 10). This was also true under regenerative management (humanure K+ $M_{treatment}$ = 174.65 [109.51, 175.75]), although one humanure plot (R09_Humanure (Block C)) was significantly lower than the other two, overlapping completely with the control and SF treatments and thus making the difference between the treatments less certain.

Fig 10 – Soil potassium after three years of cropping. The green lines show the boundaries for Index 2-, the recommended range for arable crops.

The interaction plot reveals a trend of K+ increasing under regenerative management as compared with conventional management, however looking at the raw data indicates that this trend has a low degree of certainty. Looking at in-block comparisons reveals that control treatments show an increase in K+ under regenerative management as compared with conventional management for all three Blocks, however for humanure treatment, Block A and Block C show a decrease, as does Block A for SF treatment.

Median humanure values fell within the Index 2- value range, the recommended range for arable crops [49], with some individual plots falling outside of this range. Under conventional management, the control and SF treatment plots were generally just below this range, but were elevated to be within this range under regenerative management.

3.2.9 Worms

Under conventional management, worm count was highest in the humanure treatment plots (humanure Worms $M_{treatment}$ = 13 [12, 13]), though the SF and control treatments were not considerably lower (SF Worms $M_{treatment}$ = 11 [9, 13]; control Worms $M_{treatment}$ = 11 [9, 12]) (Fig 11).

Fig 11 – Total worm count per 15cm^3 hole after three years of cropping.

Under regenerative management this difference was even more pronounced, with humanure achieving a median worm count of $M_{treatment}$ = 23 [22, 23]. These findings indicate that fertilisation type is a significant predictor of worm count, with a strong positive effect associated with humanure addition.

Worm counts were significantly higher for all treatments under regenerative management than conventional management, indicating that management type is a significant predictor of worms, with a strong positive effect associated with regenerative management. The interaction plot

suggests a possible interaction effect when combining both humanure fertiliser and management, leading to an even larger increase in worm count than would be expected from either factor alone.

3.2.10 Respiration

After three years of cultivation, the soil microbial respiration rate was 3-6 times higher than the baseline soil value for all plots (baseline respiration $M_{treatment}$ = 0.32g CO_2 kg dry soil^{-1} y^{-1} [0.30, 0.35]) (Fig 12).

Fig 12 – Soil microbial respiration rate after three years of cropping. The red line shows the baseline soil median value, and the grey lines show the baseline soil value range.

Under conventional management humanure treatment resulted in the highest respiration rate (humanure respiration $M_{treatment}$ = 1.66g CO_2 kg dry soil^{-1} y^{-1}[1.66, 1.75]), followed by SF treatment (SF respiration $M_{treatment}$ = 1.23g CO_2 kg dry soil^{-1} y^{-1} [1.09, 1.42]). The low degree of overlap indicates a significant positive effect of humanure on respiration rate under conventional management.

Under regenerative management humanure treatment also resulted in the highest respiration rate (humanure respiration $M_{treatment}$ = 1.73g CO_2 kg dry soil^{-1} y^{-1}[1.46 1.95]), but the effect is less pronounced due to a high degree of overlap with SF treatment (SF respiration $M_{treatment}$ = 1.57g CO_2 kg dry soil^{-1} y^{-1} [1.41, 1.88]).

The interaction plot reveals a positive effect of regenerative management on soil microbial respiration, although this effect is less pronounced for humanure treatment, indicating a weaker positive response to regenerative management compared with the control and SF treatments. Looking at in-block comparisons between conventional and regenerative management, Block A and B found higher values under regenerative management in two of three treatments, and Block C only one of three.

These findings suggest a positive effect of humanure and of regenerative management on soil microbial respiration rate, though the magnitude of these effects were variable.

3.2.11 Fungal biomass

Under conventional management, humanure treatment resulted in the highest median values of fungal biomass (humanure Fungi $M_{treatment}$ = 5.87µg ml^{-1} air dry soil [4.10, 6.21]). The control plots showed extreme variation (control Fungi M = 2.07µg ml^{-1} air dry soil [0.00, 15.53]) with very high values reported in plot C09_Control (Block C), obscuring any clear positive effect of humanure application (Fig 13).

Fig 13 – Soil fungal biomass, calculated from direct enumeration and measurement via light microscopy.

Under regenerative management a similar trend is observed, with humanure reporting the highest median fungal biomass, and the highest reported raw values (humanure Fungi $M_{treatment}$ = 16.18µg ml^{-1} air dry soil [12.66, 20.70]). However, there remained a significant degreed of overlap between the treatment results.

The interaction plot reveals a positive effect of regenerative management on fungal biomass. Additionally, a much lower number of null (zero) results were observed under regenerative management. Direct comparison of each fertiliser within each Block showed that M_{plot} fungal biomass was higher under regenerative management in all nine cases.

These findings indicate that management type is a significant predictor of fungal biomass, with a clear positive effect of regenerative management. The effect of fertiliser type is less clear, although humanure appears to have a promising positive effect, particularly when compared with synthetic fertiliser.

4. Discussion

4.1 Crop Growth Parameters

4.1.1 Humanure in Conventional Management

Under conventional management, humanure treatment resulted in a small increase in crop yield as compared to the unfertilised control, with an improvement of 0.34t/ha between the median values. This suggests a weakly positive fertilisation effect and is consistent with other studies which found that humanure increased crop yields compared to no fertilisation [13,-15]. However, the yield from the synthetic fertiliser (SF) treatment was higher still, improving upon the humanure treatment by an additional 0.38 t/ha. These results indicate that the direct substitution of recommended synthetic fertiliser rates with humanure does not result in equivalent crop yields within a three-year timeframe.

These results should be interpreted with caution due to small sample size and the relatively large variation observed in the SF treatment. Moreover, all reported yields were markedly lower than the 2024 Yorkshire regional average of 5.2 t/ha [50], including the synthetic fertiliser treatment, which is typically considered the “best practice” benchmark. This general underperformance raises the possibility that other environmental or management factors may have constrained yield across all treatments. One possibility is bird damage, which was noted in June. The other is the markedly wet and low-sun conditions of April and May compared to the previous five years [51], which are critical months for ear development, as well as wet weather in September which delayed harvest and could have led to yield losses from disease and rot.

Analysis of individual crop performance parameters offers further insight into these yield patterns. The humanure plots exhibited better plant establishment, greater tillering, higher grain number and higher grain mass compared to the control, which contributed to the yield increase over the control. Interestingly, while SF plots showed poorer establishment than the humanure plots, they had a

markedly higher number of grains per head, which appears to be the primary factor driving their higher overall yield. This indicates that grain number was the most significant determinant of yield under conventional management. Additional, grain loss due to damage and disease was higher in the humanure plots than the control and SF plots, and could have led to a slightly lower yield than would have been expected from the establishment and growth measures.

These findings are consistent with the AHDB Barley Growth Guide [36] which suggests that final yield is more strongly influenced by grain number than grain size. The guide also highlights a potential negative correlation between the number of grains per plant and plant density (plants/m^2), a pattern that is also observed in the present study.

Barley, particularly two-row varieties such as in the present study, is characteristically sink-limited. This means that it often has insufficient grains to store all assimilate formed during grain filling [52]. As such, early developmental processes that influence ear and grain number are especially important for yield.

Synthetic fertilisers may therefore be better equipped to support these critical early growth stages by supplying nitrogen in readily available forms at precisely the right time. This capacity for targeted nitrogen delivery offers a distinct advantage over the slower, less predictable release from organic amendments like humanure. This finding underscores the role of optimised nutrient timing and formulation in supporting high-yielding, intensively bred cereal crops in a conventionally managed system.

4.1.2 Humanure in Regenerative Management

Under regenerative management a similar trend was observed, with humanure fertilisation improving yield by 2.12t/ha compared to the unfertilised control. This substantial increase provides clear evidence that humanure can deliver meaningful fertilisation benefits to spring barley. The fact that these benefits emerged within just three years is encouraging and suggests that humanure can become agronomically effective within a relatively short time frame when managed appropriately.

Although the median yield for the synthetic fertiliser treatment was higher than that of humanure (SF $M_{treatment}$ = 4.15 t/ha [1.42, 4.94]; humanure $M_{treatment}$ = 2.94 t/ha [2.20, 3.10]), there was considerable overlap in yield values between treatments. Notably, all three humanure plots outperformed plot R04_SF in Block A, which had particularly poor performance and contributed to the high variance in the SF treatment. This overlap reduces the likelihood of a statistically significant difference between the two treatments and suggests that, under regenerative management, humanure may begin to approach the performance of synthetic fertiliser. These findings indicate the potential for humanure to serve as a viable alternative nutrient source in regenerative cropping systems without compromising yield.

Analysis of individual crop performance parameters revealed that humanure-treated plots exhibited similar establishment, tillering, and grain mass to the control. However, they showed a markedly higher number of grains per head, indicating that grain number was the key driver of yield improvement, which aligns with the findings from the conventional plots.

The synthetic fertiliser treatment once again showed lower establishment than humanure but achieved both a higher grain number per head and a greater grain mass. The lowest median grain number and grain mass values within the SF treatment were recorded in plot R04_SF — the same plot with the lowest overall yield — further substantiating the strong relationship between grain number and yield performance.

4.1.3 Comparing Conventional and Regenerative Management

Across all fertiliser treatments, the regenerative plots consistently outperformed their conventional counterparts in terms of yield. This is a particularly noteworthy result, as it challenges the prevailing narrative that conventional management delivers the highest yields, while regenerative approaches—though environmentally beneficial—typically do so at the expense of productivity.

Most strikingly, both the humanure and SF treatments achieved higher median yields under regenerative management than the SF treatment under conventional management, which is

generally regarded as a “best practice” benchmark. This result provides strong evidence that regenerative farming practices can enhance yield potential, not just environmental outcomes. It represents a compelling case for the wider adoption of regenerative agriculture in temperate cereal cropping systems.

While crop establishment was generally lower under regenerative management, this did not appear to constrain final yield. Tillering was comparable between systems, and although grain mass was lower for the control and humanure treatments under regenerative conditions, it was similar between systems for the synthetic fertiliser treatment. Overall, grain number was higher under regenerative management, reinforcing its positive impact on yield.

Interestingly, a higher incidence of diseased or damaged heads was observed in the conventional plots, which may have contributed to the lower yields. This finding is counterintuitive, given that the conventional system received fungicide applications, whereas the regenerative plots had never been treated. Although this observation could not be analysed in statistical detail, it lends anecdotal support to the idea that regenerative systems—with their greater ecological diversity—may offer a degree of disease suppression through enhanced biological resilience.

Whilst literature is inconclusive about whether organic amendments improve yields versus synthetic fertilisers [53-56] it is likely the case that the potential advantages are highly context specific. The findings of the present study support the hypothesis that humanure is better suited to regenerative systems rather than serving as a direct substitute for synthetic fertilisers in conventional systems.

4.1.4 Interaction Effects

Humanure application led to improved yields under both conventional and regenerative management, demonstrating its potential as a fertiliser across systems. However, the yield gain from humanure was notably greater under regenerative conditions compared to conventional. This suggests an interaction effect, where the benefits of humanure are amplified by the regenerative management context.

This pattern implies that humanure was more effectively utilised in regenerative systems—likely due to enhanced soil biological activity, improved structure, and reduced disturbance—all of which promote the breakdown and mineralisation of organic matter. These conditions support more efficient nutrient release and uptake, allowing crops to better capitalise on the fertiliser potential of humanure.

Crucially, this trend does not reflect a diminished performance of synthetic fertiliser under regenerative management, as yields for that treatment were also higher than in conventional plots. Instead, it highlights that regenerative practices may improve nutrient use efficiency across the board, particularly benefiting organic inputs like humanure that rely on biological processes to become plant-available.

This supports the theory that regenerative practices improve microbial functions and associated nutrient cycling [57], including the mineralisation of nutrients in organic residues as they decompose, making them plant-available [58].

This principal finding underscores the importance is a of soil health in driving plant productivity and challenging the conventional wisdom that intensive practices and chemical inputs are required for yield maximisation.

4.2 Soil Health

4.2.1 Soil Organic Matter and Carbon

Soil organic matter (SOM) and total carbon (C_{tot}) are related measures which are highly correlated [59].

After three years of application, humanure fertilisation resulted in consistently higher SOM than both the control and the SF treatment across both management systems, and raised the SOM content above that which was recorded in the baseline soil assessment at the start of the experiment. Additionally, regenerative management resulted in higher SOM than conventional

management across all three fertilisation treatments. These findings indicate that both humanure application and regenerative management have positive effects on SOM. These findings are expected, due to the direct addition of organic material to the soil, and the extensive evidence for the benefits of regenerative practices to soil carbon, such as tillage reduction [60, 61] and maintaining living roots in the soil [62, 63].

The control and SF treatments did not ever exceed the range recorded in the baseline soil assessment, indicating that they had no positive effect on SOM. This is unsurprising, since no organic matter is directly added in either case. Under conventional management both the control and SF treatments actually saw a slight reduction in SOM compared to the baseline, which supports the theory that practices like heavy tillage accelerate the breakdown of SOM and release it as CO_2 ([61, 64].

Increasing SOM offers several well-established benefits to soil health. It improves soil structure, enhances water holding capacity, helps regulate temperature, and provides both food for soil microbes and a slow-release source of nutrition for plants [24, 65, 66, 67]. These benefits collectively contribute to the long-term health and productivity of the soil ecosystem.

While the increase in SOM observed in this study is a positive indicator of broader ecological and agronomic benefits, the retention of this organic matter in the soil system is not straightforward. Soil carbon dynamics are highly complex, and pose challenges for determining changes in long-term storage versus shorter-term fluxes [68, 69]. A more detailed analysis of long-term organic matter retention and the specific carbon fractions involved would be necessary to fully understand the persistence and behaviour of the added material, which was beyond the scope of this work.

4.2.2 Water Stable Aggregates

Aggregate stability refers to a soil's resistance to structural breakdown, and is an important indicator of soil health. Low aggregate stability may indicate a soil that is more prone to erosion. Slaking, a specific type of breakdown, occurs when larger air-dry soil aggregates are suddenly immersed in

water, and the aggregates are not strong enough to withstand the internal stresses induced by rapid water uptake. This experiment looked at sudden immersion and agitation aggregates in water.

The difference in water-stable aggregates (WSA) between conventional management and regenerative management was one of the most striking and significant findings of this study, with regenerative management increasing median WSA by 12-23% across all three fertiliser treatments.

WSA is influenced by organic matter, which plays a critical role in improving aggregate stability by binding mineral particles into larger, more stable aggregates and by slowing the rate of wetting [70]. Soil ecology can also contribute to aggregate formation, with 'sticky' root exudates 'gluing' soil particles together [71] and promoting fungal growth which also bind particles together [72]. SOM, fungal biomass and microbial respiration were elevated under regenerative management, which is concordant with these expectations. This also aligns with understanding that over time repeated tillage can breakdown these aggregates [73].

Interestingly, WSA saw no clear effect of fertiliser type, despite the fact that humanure treatment is the direct addition of organic material to the soil. It is anticipated that this is due to the longer time frame required for the surface-applied SOM to be incorporated into the soil, and the slow formation of these aggregates [74, 75].

4.2.3 Nitrogen

Nitrogen dynamics are highly temporal, with inputs and transformations occurring through complex interactions between soil, plants, microbes, air, and water. Thus, these single snapshot measurements offer limited insight into nitrogen dynamics through key crop growth stages or how nitrogen cycled through the system. Instead, they offer insight only into the differences between the fertiliser treatments and management types at the time of sampling. No strong trends were observed, with the only noticeable difference being a slight reduction in both N_{tot} and N_{in} in the control plots under conventional management. Otherwise, values had high variance and exhibited

substantial overlap between treatments, reinforcing the conclusion that neither fertiliser type nor management system had a strong or detectable effect on soil nitrogen levels post-harvest.

Total nitrogen (N_{tot}) comprised <1% of the oven-dry soil mass, with only around 0.1% of it present as inorganic nitrogen (N_{in}) – the fraction readily available to plants. N_{in} is chiefly comprised of two main forms, nitrate (NO_3^-_N), which accounted for approximately 75% of N_{in}, and ammonium (NH_4^+_N), which comprised the other 25%.

N_{tot} levels remained close to the baseline soil measurements, suggesting that total nitrogen stocks (principally organic N) were largely stable over the three-year period, regardless of fertilisation and management. This is somewhat surprising, given that humanure additions contained predominantly organic N (80-90%), in contrast to SF which was entirely inorganic. One might expect some accumulation of organic nitrogen in the soil from humanure applications; however, the absence of a clear increase in N_{tot} suggests that this organic fraction was either effectively mineralised and taken up by crops or lost from the soil system, or was simply very small relative to the existing soil N pool.

N_{in} levels were considerably lower than the baseline values, however this is expected given that baseline samples were taken before cultivation, while year-three measurements were post-harvest, when residual N_{in} is at its lowest.

4.2.4 Phosphorus

Phosphorus (P) is a key macronutrient for plant growth, but many UK soils have elevated P levels due to historical over-application, leading to limited crop response to additional P and potential environmental risks [76]. As a result, current nutrient strategies aim to maintain, rather than increase, soil P by replacing only what is removed at harvest. The recommended level for cereals is P Index 2, or 16–25 mg/l plant-available P (P_{av}) [49, 77].

In this study, the baseline P_{av} was below the recommended Index 2 prior to cultivation ($M_{baseline}$ = 6.23mg/kg). If this value had remained stable after three crop harvests, it would suggest that

accumulation (via direct inputs and mineralisation of organic P) was equal to depletion (via plant uptake and losses from the system).

In the control plots under conventional management (no P input), P_{av} remained unchanged after three years, suggesting minimal P uptake—possibly explaining the poor crop performance. In contrast, regenerative control plots showed a slight Pav decline, indicating some crop utilisation.

Synthetic fertiliser (SF) plots under conventional management saw a slight P_{av} increase ($M_{treatment}$ = 8.08 mg/kg), suggesting accumulation slightly in excess of depletion. However, the resultant value remains well below the Index 2 range. Under regenerative management, P_{av} in SF treated plots remained stable, suggesting a good match between P input and crop demand.

Humanure plots under both management regimes showed significant P_{av} increases compared with the baseline and other fertiliser treatments (conventional $M_{treatment}$ = 17.97mg/kg; regenerative $M_{treatment}$ = 16.72mg/kg), reaching the target P Index 2 and indicating that accumulation significantly exceeded depletion.

Interestingly, the applied P_{av} from humanure was lower than from SF, yet the soil P_{av} increase was greater. This is likely due to the fact that a large percentage of the total P within organic material is in organic forms, and require mineralisation to become plant-available [78-80]. Thus, the total P application was likely far higher in the humanure plots than the SF plots.

Estimates suggest that 50-80% of applied organic P from manures become plant available within a growing season [49, 81, 82], and these results indicate that some of this applied organic P was successfully mineralised, leading to the elevation of soil P_{av}. It's also possible that humanure influenced mineralisation of native soil organic P, as evidence suggests that soil organic matter increases abundance of phosphorus solubilising bacteria [83].

The final P_{av} values fall within the target Index 2 range, however the large increase over the baseline values raises concerns about over-accumulation over time following repeat applications. These

concerns are well established for sewage sludge biosolids [84-87], where it is noted that most biosolids applications apply more phosphate than is taken off in the following crop [88].

It should be noted of course that humanure differs greatly from biosolids in composition, so direct comparisons should be made cautiously, and a single-season snapshot cannot fully capture the complex, dynamic transformations of P in soil systems. Future research should monitor cumulative effects of repeated applications, and should determine the relative proportions of different forms of P within the compost material.

Finally, P_{av} values were highly variable among the separate humanure plots, suggesting inconsistencies in mineralisation, uptake, or other environmental factors. This reinforces the need for a better understanding of P transformation dynamics during the composting process and within the soil system.

4.2.5 Potassium

Potassium is an essential plant macronutrient, with recommended levels for arable crops in the UK at Index 2- (121-180mg/kg [89]). Baseline K+ values were not available for comparison, limiting the ability to determine directional changes over time.

Humanure treatment elevated soil potassium (K+) levels compared to the control and SF treatments, however most humanure values remained within the recommended Index 2- range. Under regenerative management, K+ levels were higher for the control and SF plots than under conventional management, whereas humanure exhibited inconsistent trends. Humanure treatment was only higher under regenerative management than conventional management in one out of the three of the comparison plots.

Previous studies have shown that organic amendments, including manure and sewage sludge, can increase soil K^+ concentrations [90] and may act as a slow-release source of potassium, helping to

maintain more stable soil levels compared to the short-term fluxes typically associated with inorganic K fertilisers [91].

There is also evidence that such amendments not only provide nutrients, but may also enhance plant uptake of nitrogen, phosphorus, and potassium [92]. This may contribute towards explaining the yield increases observed between humanure and the control.

As with phosphorus, it remains unclear whether ongoing humanure applications would lead to excessive potassium accumulation [93], or whether the inputs are balanced by plant uptake and losses.

Again, it is difficult to directly compare the inputs of potassium which are measured in different forms. Synthetic fertiliser contains mineral potassium, often as potash (K_2O). Organic materials will contain varying quantities of mineral potassium, exchangeable potassium (held on the surface of organic matter particles and readily available by exchange with other cations) and potassium stored within organic compounds which is less readily available [91, 93, 94].

Without a clear view of potassium additions, transformations and cycling, nor of the quantity of potassium taken up into the growing crops, a snapshot view of exchangeable K+ in the soil is of limited value [95]. More detailed analysis of potassium forms and fates would be needed to determine whether observed differences were due to differences in supply, transformation, losses or uptake.

4.2.6 Worms

Earthworms are recognised as vital organisms in soil ecosystems, and their presence is often considered a good indicator of biological soil health [96, 97]. They enhance soil aeration, which improves water drainage and root penetration to foster better conditions for plant growth [98]. Earthworms are also integral to the mixing and mechanical breakdown of organic matter (OM) into

smaller fragments, enhancing contact surfaces for mineralising microorganisms [99], thus promoting nutrient cycling within the soil [100].

The notable increase in earthworm counts observed under regenerative management and in response to humanure addition suggests that these practices support favourable conditions. This is consistent with wider literature which finds that minimising physical disturbance increases earthworms, and also that organic material additions serve as a food source for worms [100-102]. Earthworms have also been found to be negatively impacted by pesticide application [103, 104], which could contribute to the difference in worm abundance between the conventional and regenerative plots.

4.2.7 Respiration

Microbial respiration is a measure of the CO_2 released from soil as a result of the metabolism of organic matter by microorganisms, for energy and biomass production [105]. More broadly, it is a coarse and simple indicator of microbial abundance and activity [106]. Soil microorganisms play a critical role in nutrient transformation processes and organic matter degradation, and many form important mutualistic associations with plant roots [107; 108].

After three years of cropping, the soil microbial respiration rate of all plots was 3-6 times higher than the baseline soil, which indicates that organic matter is being actively broken down by soil microorganisms. This process is particularly critical in the humanure fertilised plots in order to support the mineralisation of the organically bound nutrients into plant-available forms.

Under conventional management, humanure resulted in the highest microbial respiration rate of the three fertiliser treatments, indicating a positive effect. This is to be expected, as the addition of organic matter is positively correlated with microbial respiration rate [109-113]. A three year study by Iovieno et al. [111] found a positive relationship between food-waste compost and microbial activity (including respiration rate), and concluded the usefulness of repeated high-rate compost applications for soil microbial health.

Under regenerative management, the difference in respiration rate between humanure and the other fertiliser treatments was less pronounced, but the respiration rates for all three were elevated in comparison to the conventionally managed plots. This indicates a positive effect of regenerative management on microbial respiration rate. This is consistent with findings that tillage reduces overall microbial abundance [107, 114], despite evidence of short initial bursts of CO_2 efflux immediately following tillage due to aeration and exposure of previously unavailable OM [115; 116]. Additionally, the use of cover crops and diverse crop production regimes have been shown to increase abundance, activity and diversity of soil microbes [117, 118]. Singh et al. [119] investigated the effect of multiple regenerative practices on bacterial community structure over a 3 and 5-year experiment and found they were all effective in improving bacterial community structure and overall soil health.

4.2.8 Fungi

Fungi are important indicators of biological soil health due to their ubiquity, diversity and sensitivity to environmental change [120]. This diverse kingdom plays key roles in organic matter decomposition, nutrient cycling, plant pathogen suppression, and carbon sequestration [121, 122].

In this study, no clear effect of fertiliser type on fungal biomass was detected. However, there was some indication of a positive effect from regenerative management practices compared to conventional management. This aligns with existing literature, which highlights the detrimental effects of tillage on fungal communities [114] and the beneficial impact of cover cropping [123].

That said, the data should be interpreted with caution. Fungal biomass measurements exhibited considerable variability, even among triplicates taken from the same sample bag. This high within-treatment variation limits the reliability of the results and raises concerns about the suitability of the method for this type of analysis. These findings highlight a broader challenge in soil biology: the difficulty of obtaining accurate and reproducible estimates of fungal biomass using simple and cost-

effective approaches. To improve confidence in future results, either alternative, more robust methods should be adopted, or the number of true replicates should be substantially increased.

4.3 Composition and Use of Humanure

The composition of the three humanure batches varied considerably (Supporting Information S2), as expected for materials produced under different conditions [7, 124]. While total nitrogen (N_{tot}) was comparable across batches, quantities and forms of inorganic nitrogen (N_{in}) differed significantly, reflecting the complex and variable nature of N transformation during storage. Despite this, C:N ratios (13.59-16.07) aligned closely with the expected range for mature composts [125, 126].

High within-batch variability further illustrates the heterogeneity of homemade humanure and the challenge of achieving uniform nutrient content. This complicates accurate dosing for scientific research and poses a barrier to potential commercial applications, where standardisation may be required. Continued reporting of humanure composition from different systems is therefore vital to help establish reliable reference ranges.

In this study, a matched N_{tot} approach was taken between humanure and synthetic fertiliser (SF). This is a common approach [7], and the quantities of humanure that would have been required to match N_{in} would have been infeasible. However, organic N mineralises slowly, and estimates vary for the quantity which becomes plant-available in each growing season. A review by Rigby et al. [127] suggests this value falls between 2-24.5% for composted biosolids, whilst research on animal manures suggests a range of -2% to 48%, with plant-available nitrogen (PAN) generally showing an inverse relationship with C:N ratio [128]. A value of 10% PAN is sometimes adopted as a guideline figure [127], with an understanding that smaller proportions continue to be mineralised in subsequent years, contributing to a long-term residual effect [129].

The slow mineralisation of organic N introduces uncertainty in nutrient supply, both for scientific comparison and for practical use. Future studies might better align fertiliser equivalency by matching

PAN rather than N_{tot}, though this approach requires careful year-on-year adjustment to account for ongoing mineralisation.

5. Conclusion

This study explored the impacts of applying humanure as a fertiliser in both conventional and regenerative cereal production systems. While findings are preliminary due to the small sample sizes, they provide useful insights into how humanure interacts with different management practices, and where it may best fit into future agricultural strategies.

In conventional systems, humanure showed potential as a direct replacement for synthetic fertiliser. Although crop performance under humanure was slightly lower overall, a high degree of overlap in the data suggests that these differences may not be statistically significant. This reduction in yield was likely driven by a lower number of grains per head, potentially linked to limited nitrogen availability early in the growing season due to the slower nutrient release profile of humanure. However, humanure treatments led to notably higher biological indicators—specifically microbial respiration and earthworm abundance—compared to both synthetic fertiliser treatment and the control. Physical soil characteristics showed little meaningful variation across treatments, while soil organic matter, available phosphorus, and potassium were all elevated under humanure application. Total and inorganic soil nitrogen were similar under humanure and SF application, and higher than the control.

The observed soil health benefits under humanure fertilisation may take longer than three years to confer any benefit to crop production, with previous studies indicating that it may take longer than three years for organic production to 'catch up' to conventional production [130]. More long term studies are sorely needed within this area of research to fully model this transition time. Practical challenges also remain for direct substitution at farm scale, including the large quantities of humanure required, logistical issues related to transport and application, and the difficulty in

delivering a standardised product, both for informed nutrient management and to guarantee product safety.

Under the regenerative system, humanure performed particularly well. It was hypothesised that the enhanced biological activity associated with regenerative practices would better facilitate nutrient cycling from the organic inputs, and the results support this. Yields under regenerative humanure treatment exceeded both the regenerative control and the conventional humanure and synthetic fertiliser treatments, due mainly to increased grain number and a reduction in damage and disease-related losses. Regenerative-humanure treatment also saw the highest SOM content, microbial respiration rates, and earthworm counts across all plots, indicating a positive interaction effect.

This is a notable finding, especially after the review by Allen et al. [7] highlighted a lack of reporting on biological and physical soil health parameters in studies using faecal-based amendments. By assessing these indicators, this study begins to fill that gap and suggests that the combination of regenerative management and organic waste amendments like humanure may provide synergistic benefits for both soil and crop outcomes.

Moreover, many previous studies have assessed humanure within single year, isolated frameworks; this study’s design allowed for a side-by-side comparison across two contrasting systems over a three-year period, revealing for the first time clear differences in fertiliser performance depending on the management context.

Additionally, regenerative practices consistently outperformed conventional management across all fertiliser treatments for most crop performance indicators, as well as SOM, water-stable aggregates, and biological soil health. This suggests that regenerative management alone may improve soil function and productivity, challenging the idea that conventional management should be the default benchmark. This insight has major implications for the way we model and design waste reuse systems: rather than focusing solely on input substitution, we may need to rethink the management context entirely to best match resources with appropriate land management contexts.

Macronutrient dynamics were a key point of discussion; under both management regimes, humanure elevated soil available phosphorus (P_{av}) and potassium (K+) levels relative to both the controls and the synthetic fertiliser treatments, whilst the effect of management type alone was comparatively minor. Though elevated, P_{av} and K+ levels under humanure application remained within agronomic target ranges (Index 2) and thus posed no immediate concern for crop safety. However, continued applications at these rates could risk long-term over accumulation, and warrants further monitoring and research.

Finally, it is important to recognise the limitations of the study. Sample sizes were small—a common constraint in plot-scale agricultural experiments—and this restricts the statistical power of the results. Trends discussed here should be considered exploratory, rather than definitive. Nonetheless, the observed patterns offer promising directions for future research.

Taken together, these findings suggest that humanure holds promise as a fertiliser input, particularly within regenerative systems where biological activity can support decomposition and nutrient mineralisation of the material. This supports existing findings of positive fertility and soil health benefits of sewage sludge biosolids, compared with synthetic fertiliser [26, 131, 132]. Both the integration of recycled organic waste streams into agricultural production, and the transition towards more regenerative production systems, could help to reduce dependence on synthetic fertilisers whilst also improving soil health and closing nutrient loops. With appropriate safeguards and further practical considerations, these approaches are well positioned to support a more sustainable and circular farming system, without compromising productivity.

6. References

1. Berendes DM, Yang PJ, Lai A, Hu D, Brown J. Estimation of global recoverable human and animal fecal biomass. Nat Sustain. 2018;1(11):679-685. doi: 10.1038/s41893-018-0167-0. PMID: 38464867; PMCID: PMC10922008.

2. WHO & UNICEF. Progress on household drinking water, sanitation and hygiene 2000–2022: special focus on gender. New York (NY): United Nations Children's Fund (UNICEF) and World Health Organization (WHO); 2023.

3. Webb P, Benton TG, Beddington J, Flynn D, Kelly NM, Thomas SM. The urgency of food system transformation is now irrefutable. Nat Food. 2020 Oct;1(10):584-585. doi: 10.1038/s43016-020-00161-0. PMID: 37128102.

4. Menegat S, Ledo A, Tirado R. Greenhouse gas emissions from global production and use of nitrogen synthetic fertilisers in agriculture. Sci Rep. 2022 Aug 25;12(1):14490. doi: 10.1038/s41598-022-18773-w. Erratum in: Sci Rep. 2022 Nov 17;12(1):19777. doi: 10.1038/s41598-022-24242-1. PMID: 36008570; PMCID: PMC9411506.

5. Alexander P, Arneth A, Henry R, Maire J, Rabin S, Rounsevell MDA. High energy and fertilizer prices are more damaging than food export curtailment from Ukraine and Russia for food prices, health and the environment. Nat Food. 2023 Jan;4(1):84-95. doi: 10.1038/s43016-022-00659-9. Epub 2022 Dec 23. PMID: 37118577.

6. Agricultural and Food Policy Centre. Economic Impact of Higher Fertilizer Prices on AFPC's Representative Crop Farms. College Station (TX): Agricultural and Food Policy Centre, Texas A&M University. 2022.

7. Allen K, Rodríguez López EL, Banwart SA, Evans B. A Systematic Review of the Effects of Fecal Sludge Derived Amendments on Crop Growth and Soil Health. ACS ES&T Engg. 2023 May;3(6):746-761. doi: 10.1021/acsestengg.2c00438

8. Trimmer JT, Margenot AJ, Cusick RD, Guest JS. Aligning Product Chemistry and Soil Context for Agronomic Reuse of Human-Derived Resources. Environ Sci Technol. 2019 Jun 4;53(11):6501-6510. doi: 10.1021/acs.est.9b00504. Epub 2019 May 2. PMID: 31017776.

9. Witek-Krowiak A, Gorazda K, Szopa D, Trzaska K, Moustakas K, Chojnacka K. Phosphorus recovery from wastewater and bio-based waste: an overview. Bioengineered. 2022 May;13(5):13474-13506. doi: 10.1080/21655979.2022.2077894. PMID: 36700471; PMCID: PMC9275867.

10. Patil A, Sangami S, Chandak PG. Zero waste toilet a sensor-operated urine diverting toilet for sustainable sanitation and fertilizer production. IJoSI. 2024 Jun;8(2):70-86. doi: 10.6977/IJoSI.202406_8(2).0006

11. Couder L, Kibuthu, S. Unlocking the circular economy potential to tackle the sanitation challenge. Field Actions Sci. Rep. 2020 Dec;22:72-77.

12. Jenkins JC. The Humanure Handbook: A Guide to Composting Human Manure, Emphasizing Minimum Technology and Maximum Hygienic Safety. Jenkins Publishing; 1994.

13. Uju EU, Ekpe II, Ihem EE, Mgbeahuru CI, Isaiah AA. Impact of humanure on soil properties and yield of watermelon (citrulluslanatus) in Imo state Nigeria. AGRIJ. 2022;1(1);71-86.

14. Torgbo S, Quaye EA, Adongo TA, Opoku N. The Effects of Dried Faecal Sludge and Municipal Waste Co-Compost on Microbial Load and Yield of Cabbage (Brassica oleracea L. var. capitata) and Lettuce (Lactuca sativa). JMBFS. 2018;7(6);555-561.

15. Ekpe II, Ihemtuge SC, Okoye C, Ahukaemere CM, Onuora MD, Okere SE, et al. Effect of Humanure and Rumen Digesta on Soil Physico-Chemical Properties and the Yield of Cucumber in Imo South-Eastern, Nigeria. Futo Journal Series. 2017; 3(1);13-26.

16. Sumberg J, Giller KE. What is 'conventional' agriculture? Glob Food Sec. 2022; 32;100617. doi: 10.1016/j.gfs.2022.100617

17. Newton P, Civita N, Frankel-Goldwater L, Bartel K, Johns C. What Is Regenerative Agriculture? A Review of Scholar and Practitioner Definitions Based on Processes and Outcomes. Front. Sustain. Food Syst. 2020; 4; 577723. doi: 10.3389/fsufs.2020.577723

18. Yi J, Zeng Q, Mei T, Zhang S, Li Q, Wang M, et al. Disentangling drivers of soil microbial nutrient limitation in intensive agricultural and natural ecosystems. Sci Total Environ. 2022 Feb 1;806(Pt 1):150555. doi: 10.1016/j.scitotenv.2021.150555. Epub 2021 Sep 24. PMID: 34844329.

19. Ishaq SL. Plant-microbial interactions in agriculture and the use of farming systems to improve diversity and productivity. AIMS Microbiol. 2017 May 11;3(2):335-353. doi: 10.3934/microbiol.2017.2.335. PMID: 31294165; PMCID: PMC6605018.

20. Bhattacharyya SS, Furtak, K. Soil–Plant–Microbe Interactions Determine Soil Biological Fertility by Altering Rhizospheric Nutrient Cycling and Biocrust Formation. Sustainability. 2022 Dec 29;15(1):625.

21. Tsiafouli MA, Thébault E, Sgardelis SP, de Ruiter PC, van der Putten WH, Birkhofer K, et al. Intensive agriculture reduces soil biodiversity across Europe. Glob Chang Biol. 2015 Feb;21(2):973-85. doi: 10.1111/gcb.12752. Epub 2014 Nov 17. PMID: 25242445.

22. Chiriac OP, Pittarello M, Moretti B, Zavattaro L. Factors influencing nitrogen derived from soil organic matter mineralisation: Results from a long-term experiment. Agric Ecosyst Environ. 2025 Apr 1;381:109444.

23. Marchuk S, Tait S, Sinha P, Harris P, Antille DL, McCabe BK. Biosolids-derived fertilisers: A review of challenges and opportunities. Sci Total Environ. 2023 Jun 1;875:162555. doi: 10.1016/j.scitotenv.2023.162555. Epub 2023 Mar 6. PMID: 36889394.

24. Lal R. Soil organic matter and water retention. Agron J. 2020 May 7;112(5):3265-77

25. Minasny B, McBratney AB. Limited effect of organic matter on soil available water capacity. Eur J Soil Sci. 2017 Oct 6;69(1);39-47.

26. Ippolito JA, Ducey TF, Diaz K, Barbarick KA. Long-term biosolids land application influences soil health. Sci Total Environ. 2021 Oct 15;791:148344. doi: 10.1016/j.scitotenv.2021.148344. Epub 2021 Jun 8. PMID: 34412404.

27. Banerjee MR, Burton DL, Depoe S. Impact of sewage sludge application on soil biological characteristics. Agric Ecosyst Environ. 1997 Dec 15;66(3):241-9.

28. Ros M, Klammer S, Knapp B, Aichberger K, Insam H. Long-term effects of compost amendment of soil on functional and structural diversity and microbial activity. Soil Use Manag. 2006 Apr 7;22(2):209-18.

29. Honvault N, Houben D, Lebrun M, Vedere C, Nobile C, Guidet J, et al. Positive or neutral effects of biochar-compost mixtures on earthworm communities in a temperate cropping system. Appl Soil Ecol. 2023 Feb;182:104684.

30. Sangare D, Sawadogo B, Sou Dakoure M, Ouedraogo DMS, Hijikata N, Yacouba H, et al. Ecological sanitation products reuse for agriculture in Sahel: effects on soil properties. Soil Discuss (preprint). 2015 Mar 31;2:291-322.

31. Pradhan SK, Cofie O, Nikiema J, Heinonen-Tanski H. Fecal Sludge Derived Products as Fertilizer for Lettuce Cultivation in Urban Agriculture. Sustainability. 2019 Dec 11;11(24): 7101.

32. Moya B, Parker A, Sakrabani R, Mesa B. Evaluating the Efficacy of Fertilisers Derived from Human Excreta in Agriculture and Their Perception in Antananarivo, Madagascar. Waste Biomass Valori. 2017 Oct 27; 10:941-52.

33. Giller KE, Hijbeek R, Andersson JA, Sumberg J. Regenerative Agriculture: An agronomic perspective. Outlook Agric. 2021 Mar;50(1):13-25. doi: 10.1177/0030727021998063. Epub 2021 Mar 2. PMID: 33867585; PMCID: PMC8023280.

34. Moebius-Clune BN, Moebius-Clune DJ, Gugino BK, Idowu OJ, Schindelbeck RR, Ristow AJ, et al. Comprehensive Assessment of Soil Health. The Cornell Framework. Third Edition. New York: Cornell University School of Integrative Plant Science; 2017 Jun.

35. AHDB. Wheat Growth Guide. Coventry: AHDB Cereals & Oilseeds; 2023a.

36. AHDB. Barley Growth Guide. Coventry: AHDB Cereals & Oilseeds; 2023b.

37. Jones A. Practical Skills in Environmental Sciences. London: Pearson; 1999.

38. Vidacycle. Slake (Wet Aggregate Stability). 2020 [cited 08/05/2025]. Available from: https://soils.vidacycle.com/soil-tests/1-2-slake-0-8/

39. Rowell DL. Soil Science Methods and Application. London: Longman. 1994.

40. British Standards. BS 1377: Part 3: 1990. Methods of test for Soils for civil engineering purposes Part 3. British Standards; 1996.

41. Pella E. Elemental organic analysis. Part 1, Historical developments. Am Lab. 1990a;22(2):116-25.

42. Pella E. Elemental organic analysis. Part 2: State of the art. Am Lab. 1990b;22(12):28-32.

43. Carter MR, Gregorich EG. Soil Sampling Methods and Analysis. 2nd ed. Canada: CRC Press; 2008.

44. Williams BL, Buttler A, Grosvernier P. The fate of ammonium nitrate added to Sphagnum Magellanicum carpets at South European mire sites. Biogeochemistry. 1999; 45:73-93.

45. Robertson GP, Sollins P, Ellis BG, Lajtha K. Exchangeable Ions, pH and cation exchange capacity. In: Robertson GP, Coleman DC, Bledsoe CS, Sollins P, editors. Standard Soil Methods for Long-Term Ecological Research. New York: Oxford University Press Inc; 1999, pp. 107.

46. Olsen SR, Cole CV, Watanabe FS, Dean LA. Estimation of Available Phosphorus in Soils by Extraction with Sodium Bicarbonate. U. S. Department of Agriculture Circular No. 939; 1954.

47. Cappo KA, Blume LJ, Raab GA, Bartz JK, Engles JL. Analytical Methods Manual for Direct/Delayed Response Project Soil Survey EPA/600/8-87/020). NV: US Environmental Protection Agency; 1987.

48. Limousin G, Tessier D, with reply from C. E. Johnson. Letters to the Editor, Eur J Soil Sci. 2003;54:641–647. doi: 10.1046/j.1365-2389.2003.00534.x.

49. AHDB. Nutrient Management Guide (RB209). Section 4 – Arable Crops. Coventry: Agriculture and Horticulture Development Board. 2023c.

50. DEFRA. Provisional cereal and oilseed production estimates for England 2024. Dataset. 2025 Jan 7 [downloaded 09/05/2025]. Available from: https://www.gov.uk/government/statistics/cereal-and-oilseed-rape-production/provisional-cereal-and-oilseed-production-estimates-for-england-2024

51. MET Office. MET office historical data from Bradford weather station. Dataset. 2025. [downloaded 09/05/2025]. Available from: https://www.metoffice.gov.uk/research/climate/maps-and-data/historic-station-data

52. Bingham IJ, Blake J, Foulkes MJ, Spink J. Is barley yield in the UK sink limited?: II. Factors affecting potential grain size. Field Crops Res. 2007 Mar 5;101(2):212-20. https://doi.org/10.1016/j.fcr.2006.11.004

53. Cai A, Xu M, Wang B, Zhang W, Liang G, Hou E, et al. Manure acts as a better fertilizer for increasing crop yields than synthetic fertilizer does by improving soil fertility. Soil Till Res. 2019 Jun;189:168-75.

54. Edmeades DC. The long-term effects of manures and fertilisers on soil productivity and quality: a review. Nutr Cycl Agroecosystems. 2003 Jun;66:165-80.

55. Hepperly P, Lotter D, Ulsh CZ, Seidel R, Reider C. Compost, Manure and Synthetic Fertilizer Influences Crop Yields, Soil Properties, Nitrate Leaching and Crop Nutrient Content. Compost Sci Util. 2009; 17(2):117-26. https://doi.org/10.1080/1065657X.2009.10702410

56. Zhang X, Fang Q, Zhang T, Ma W, Velthof GL, Hou Y, et al. Benefits and trade-offs of replacing synthetic fertilizers by animal manures in crop production in China: A meta-analysis. Glob Chang Biol. 2020 Feb;26(2):888-900. doi: 10.1111/gcb.14826. Epub 2019 Oct 26. PMID: 31495039.

57. Khangura R, Ferris D, Wagg C, Bowyer J. Regenerative Agriculture – A Literature Review on the Practices and Mechanisms Used to Improve Soil Health. Sustainability. 2023 Jan 27; 15(3):2338. https://doi.org/10.3390/su15032338

58. Kaur T, Himshikha, Singh A, Brar, SK, Kaur S, Kaur J. Enhancing Nutrient Recycling Through Regenerative Practices Under Different Agroecosystems. In: KumarS, Meena RS, Sheoran P, Jhariya MK, editors. Regenerative Agriculture for Sustainable Food Systems. Singapore: Springer; 2024 Oct 25. https://doi.org/10.1007/978-981-97-6691-8_9

59. Richardson GS, Ruark MD, Radatz T, Radatz A, Cooley E, Silva EM, et al. The influence of inherent soil factors and agricultural management on soil organic matter. Ecosphere. 2023 Mar 13;14(3):e4459.

60. Haddaway NR, Hedlund K, Jackson LE, Kätterer T, Lugato E, Thomsen IK. How does tillage intensity affect soil organic carbon? A systematic review. Environ Evid. 2017 Dec 18;6:30.

61. Wang H, Wang S, Yu Q, Zhang Y, Wang R, Li J, et al. No tillage increases soil organic carbon storage and decreases carbon dioxide emission in the crop residue-returned farming system. J Environ Manage. 2020 May 1;261:110261. doi: 10.1016/j.jenvman.2020.110261.

62. Rosolem CA, Li Y, Garcia RA. Soil carbon as affected by cover crops under no-till under tropical climate. Soil Use Manag. 2016 Nov 29;32(4):495-503.

63. Burgess PJ, Harris J, Graves AR, Deeks LK. Regenerative Agriculture: Identifying the Impact; Enabling the Potential. Report for SYSTEMIQ. Bedfordshire: Cranfield University; 2019 May 17.

64. Reicosky DC, Dugas WA, Torbert HA. Tillage-induced soil carbon dioxide loss from different cropping systems. Soil Till Res. 1997 Mar;41(1-2):105-118.

65. Bronick CJ, Lal R. Soil structure and management: a review. Geoderma. 2005 Jan;124(1-2):3-22. https://doi.org/10.1016/j.geoderma.2004.03.005

66. Tiessen H, Cuevas E, Chacon P. The role of soil organic matter in sustaining soil fertility. Nature. 1994 Oct 27;371:783-5.

67. Mohammadi K, Heidari G, Khalesro S, Sohrabi Y. Soil management, microorganisms and organic matter interactions: A review. Afr J Biotechnol. 2011 Dec 30;10(84):19840-9. DOI: 10.5897/AJBX11.006

68. Post WM, Izaurralde RC, Mann LK, Bliss N. Monitoring and Verifying Changes of Organic Carbon in Soil. Clim Change. 2001 Oct;51:73-99.

69. Jandl R, Rodeghiero M, Martinez C, Cotrufo MF, Bampa F, van Wesemael B, et al. Current status, uncertainty and future needs in soil organic carbon monitoring. Sci Total Environ. 2014 Jan 15;468-469:376-83. doi: 10.1016/j.scitotenv.2013.08.026. Epub 2013 Sep 13. PMID: 24041605.

70. Sonsri K, Watanabe A. Insights into the formation and stability of soil aggregates in relation to the structural properties of dissolved organic matter from various organic amendments. Soil Till Res. 2023 Aug;232:105774.

71. Traoré O, Groleau-Renaud V, Plantureux S, Tubeiled A, Boeuf-Tremblay V. Effect of root mucilage and modelled root exudates on soil structure. Eur J Soil Sci. 2008 Jul 7;51(4):575-81. https://doi.org/10.1111/j.1365-2389.2000.00348.x

72. Baumert VL, Vasilyeva NA, Vladimirov AA, Meier IC, Kögel-Knabner I, Mueller CW. Root Exudates Induce Soil Macroaggregation Facilitated by Fungi in Subsoil. Front Environ Sci. 2018 Nov 27;6. https://doi.org/10.3389/fenvs.2018.00140

73. Weidhuner A, Hanauer A, Krausz R, Crittenden SJ, Gage K, Sadeghpour A. Tillage impacts on soil aggregation and aggregate-associated carbon and nitrogen after 49 years. Soil Till Res. 2021 Spr;208:104878. https://doi.org/10.1016/j.still.2020.104878

74. Guest EJ, Palfreeman LJ, Holden J, Chapman PJ, Firbank LG, Lappage MG, et al. Soil macroaggregation drives sequestration of organic carbon and nitrogen with three-year grass-clover

leys in arable rotations. Sci Total Environ. 2022 Dec 15;852:158358. doi: 10.1016/j.scitotenv.2022.158358. Epub 2022 Aug 29. PMID: 36049686.

75. Amelung W, Meyer N, Rodionov A, Knief C, Aehnelt M, Bauke SL, et al. Process sequence of soil aggregate formation disentangled through multi-isotope labelling. Geoderma. 2023 Jan 1;429:116226.

76. AHDB. Phosphorus and potassium requirements of cereals. Nottingham: AHDB Horticulture. 2001 Jan 1. Available from: https://ahdb.org.uk/phosphorus-and-potassium-requirements-of-cereals

77. Knight S, Morris N, Goulding K, Johnston J, Poulton P, Philpott H. Project Report No. 529. Identification of critical soil phosphate (P) levels for cereal and oilseed rape crops on a range of soil types. Nottingham: AHDB Horticulture. 2014 Mar. Available from: https://projectblue.blob.core.windows.net/media/Default/Research%20Papers/Cereals%20and%20Oilseed/pr529-final-project-report.pdf

78. Tuszynska A, Czerwionka K, Obarska-Pempkowiak H. Phosphorus concentration and availability in raw organic waste and post fermentation products. J Environ Manage. 2021 Jan 15;278(Pt 2):111468. doi: 10.1016/j.jenvman.2020.111468. Epub 2020 Nov 2. PMID: 33152546.

79. Gagnon B, Demers I, Ziadi N, Chantigny MH, Parent LE, Forge TA, et al. Forms of phosphorus in composts and in compost-amended soils following incubation. Can J Soil Sci. 2012 Aug 1;92:711-21.

80. Lanno M, Kriipsalu M, Shanskiy M, Silm M, Kisand A. Distribution of Phosphorus Forms Depends on Compost Source Material. Resources. 2021 Oct 8;2021(10):102. https://doi.org/10.3390/resources10100102

81. Kaiser DE, Pagliari P. Understanding phosphorus fertilizers. University of Minnesota Extension. 2018. Available from: https://extension.umn.edu/phosphorus-and-potassium/understanding-phosphorus-fertilizers

82. NRM. Technical Information. Advice Sheet 24: Analysis of Manures, Slurries and Dirty Waters. Bracknell: NRM; 2021 Aug 11. Available from: https://cawood.co.uk/wp-content/uploads/2022/04/Manures-Slurries-and-Dirty-Waters-Technical-Information.pdf

83. Azziz G, Bajsa N, Naghjou T, Taulé C, Valverde A, Igual J, et al. Abundance, diversity and prospecting of culturable phosphate solubilizing bacteria on soils under crop–pasture rotations in a no-tillage regime in Uruguay. Appl. Soil Ecol. 2012;61:320–6. doi: 10.1016/j.apsoil.2011.10.004

84. Silva-Leal JA, Pérez-Vidal A, Torres-Lozada P. Effect of biosolids on the nitrogen and phosphorus contents of soil used for sugarcane cultivation. Heliyon. 2021 Mar 1;7(3):e06360. doi: 10.1016/j.heliyon.2021.e06360. PMID: 33732921; PMCID: PMC7937669.

85. Penn CJ, Sims JT. Phosphorus forms in biosolids-amended soils and losses in runoff: effects of wastewater treatment process. J Environ Qual. 2002 Jul-Aug;31(4):1349-61. doi: 10.2134/jeq2002.1349. PMID: 12175056.

86. Maguire RO, Sims JT, Coale FJ. Phosphorus Solubility in Biosolids-Amended Farm Soils in the Mid-Atlantic Region of the USA. J Environ Qual. 2000 Jul 1;29(4):1225-33.

87. Shober AL, Sims JT. Phosphorus restrictions for land application of biosolids: current status and future trends. J Environ Qual. 2003 Nov-Dec;32(6):1955-64. doi: 10.2134/jeq2003.1955. PMID: 14674517.

88. AHDB. Nutrient Management Guide (RB209). Section 2 – Organic Materials. Coventry: Agriculture and Horticulture Development Board. 2023d.

89. Potash Development Association. Soil Analysis: Key to Nutrient Management Planning. York: Potash Development Association. 2011 Nov. Available from: https://www.pda.org.uk/wp/wp-content/uploads/2015/12/PDA-lf24.pdf

90. Achkir A, Aouragh A, El Mahi M, Lotfi EM, Labjar N, Bouch MEL, et al. Implication of sewage sludge increased application rates on soil fertility and heavy metals contamination risk. Emerg Contam. 2023 Mar;9(1):100200. https://doi.org/10.1016/j.emcon.2022.100200

91. Ndoung OCN, Souza LR, Fachini J, Leão TP, Sandri D, Figueiredo CC. Dynamics of potassium released from sewage sludge biochar fertilizers in soil. J Environ Manage. 2023 Nov 15;346:119057. doi: 10.1016/j.jenvman.2023.119057. Epub 2023 Sep 22. PMID: 37742559.

92. Antoniadis V, Koutroubas SD, Fotiadis S. Nitrogen, Phosphorus, and Potassium Availability in Manure- and Sewage Sludge–Applied Soil. Commun Soil Sci Plant Anal. 2015 Jan 8;46(3):393-404.

93. Kandil EE, Abdelsalam NR, Mansour MA, Ali HM, Siddiqui MH. Potentials of organic manure and potassium forms on maize (Zea mays L.) growth and production. Sci Rep. 2020 May 29;10(1):8752. doi: 10.1038/s41598-020-65749-9. PMID: 32472061; PMCID: PMC7260245.

94. Bhattacharyya P, Chakrabarti K, Chakraborty A, Nayak DC, Tripathy S, Powell MA. Municipal waste compost as an alternative to cattle manure for supplying potassium to lowland rice. Chemosphere. 2007 Jan;66(9):1789-93. doi: 10.1016/j.chemosphere.2006.07.062. Epub 2006 Sep 7. PMID: 16956645.

95. Vantha T, Subbarayappa CT. Forms of Potassium and its Relationship with different Soil Properties from Selected Land use Systems in Southern Transect of Bengaluru. Mysore J Agric Sci. 2022;56(3):290-9.

96. Fründ, HC, Graefe U, Tischer S. Earthworms as Bioindicators of Soil Quality. In: Karaca A, editor. Biology of Earthworms. Soil Biology, vol 24. Berlin: Springer. 2010 Jan 1;261-78. https://doi.org/10.1007/978-3-642-14636-7_16

97. Alves PRL, Bandeira FO, Hennig TB. Chapter 4 - Ecological Role of Earthworms as Bioindicators of Soil Health. In: Pal Vig A, Singh J, Suthar S, editors. Earthworm Engineering and Applications. New York: Nova Science Publishers. 2022; 51-82.

98. Edwards CA, Arancon NQ. Earthworms, Soil Structure, Fertility, and Productivity. In: Biology and Ecology of Earthworms. New York: Springer. 2022 Apr 28; 303-34 https://doi.org/10.1007/978-0-387-74943-3_10

99. Lemitri A, Colinet G, Alabi T, Cluzeau D, Zirbes L, Haubruge E, et al. Impacts of earthworms on soil components and dynamics. A review. Biotechnol Agron Soc Environ. 2014;18(1):121-33.

100. van Vliet PCJ, van der Stelt B, Rietberg PI, de Goede RGM. Effects of organic matter content on earthworms and nitrogen mineralization in grassland soils. Eur J Soil Biol. 2007;43(1):222-9.

101. Hendrix PF, Mueller BR, Bruce RR, Langdale GW, Parmelee RW. Abundance and distribution of earthworms in relation to landscape factors on the Georgia Piedmont, U.S.A. Soil Biol Biochem. 1992 Dec;24(12):1357-61.

102. Scown J, Baker G. The influence of livestock dung on the abundance of exotic and native earthworms in a grassland in south-eastern Australia. Eur J Soil Biol. 2006 Nov;42(1):310-5.

103. Pelosi C, Barot S, Capowiez Y, Hedde M, Vandenbulcke F. Pesticides and earthworms. A review. Agron Sustain Dev. 2013 May 16;34:199-228.

104. Datta S, Singh J, Singh S, Singh J. Earthworms, pesticides and sustainable agriculture: a review. Environ Sci Pollut Res Int. 2016 May;23(9):8227-43. doi: 10.1007/s11356-016-6375-0. Epub 2016 Mar 7. PMID: 26951221.

105. Gunina A, Kuzyakov Y. From energy to (soil organic) matter. Glob Chang Biol. 2022 Apr;28(7):2169-2182. doi: 10.1111/gcb.16071. Epub 2022 Jan 17. PMID: 34978126.

106. Irving D, Bakhshandeh S, Tran TKA, McBratney AB. A cost-effective method for quantifying soil respiration. Soil Secur. 2024;16:100162.

107. Kraut-Cohen J, Zolti A, Shaltiel-Harpaz L, Argaman E, Rabinovich R, Green SJ, Minz D. Effects of tillage practices on soil microbiome and agricultural parameters. Sci Total Environ. 2020 Feb 25;705:135791. doi: 10.1016/j.scitotenv.2019.135791. Epub 2019 Nov 27. PMID: 31810706.

108. Zhang R, Vivanco JM, Shen Q. The unseen rhizosphere root-soil-microbe interactions for crop production. Curr Opin Microbiol. 2017 Jun;37:8-14. doi: 10.1016/j.mib.2017.03.008. Epub 2017 Apr 21. PMID: 28433932.

109. Rui Y, Murphy DV, Wang X, Hoyle FC. Microbial respiration, but not biomass, responded linearly to increasing light fraction organic matter input: Consequences for carbon sequestration. Sci Rep. 2016 Oct 18;6:35496. doi: 10.1038/srep35496. PMID: 27752083; PMCID: PMC5067501.

110. Schnürer J, Clarholm M, Rosswall T. Microbial biomass and activity in an agricultural soil with different organic matter contents. Soil Biol Biochem. 1985;17(5):611-8.

111. Iovieno P, Morra L, Leone A, Pagano L, Alfani A. Effect of organic and mineral fertilizers on soil respiration and enzyme activities of two Mediterranean horticultural soils. Biol Fertil Soils. 2009;45:555-61. https://doi.org/10.1007/s00374-009-0365-z

112. Lai L, Wang J, Tian Y, Zhao X, Jiang L, Chen X, Gao Y, Wang S, Zheng Y. Organic matter and water addition enhance soil respiration in an arid region. PLoS One. 2013 Oct 18;8(10):e77659. doi: 10.1371/journal.pone.0077659. PMID: 24204907; PMCID: PMC3799695.

113. Pérez-Piqueres A, Edel-Hermann V, Alabouvette C, Steinberg C. Response of soil microbial communities to compost amendments. Soil Biol Biochem. 2006;38(3):460-70.

114. Chen H, Dai Z, Veach AM, Zheng J, Xu J, Schadt CW. Global meta-analyses show that conservation tillage practices promote soil fungal and bacterial biomass. Agric Ecosyst Environ. 2020 May 1;293:106841.

115. Fiedler SR, Leinweber P, Jurasinski G, Eckhardt KU, Glatzel S. Tillage-induced short-term soil organic matter turnover and respiration. SOIL. 2016;2(3):475-86.

116. Jinbo Z, Changchun S, Shenmin W. Short-term dynamics of carbon and nitrogen after tillage in a freshwater marsh of northeast China. Soil Till Res. 2008 Jun;99(2):149-57.

117. Kim N, Zabaloy MC, Guan K, Villamil MB. Do cover crops benefit soil microbiome? A meta-analysis of current research. Soil Biol Biochem. 2020 Mar;142:107701.

118. King AE, Hofmockel KS. Diversified cropping systems support greater microbial cycling and retention of carbon and nitrogen. Agric Ecosyst Environ. 2017 Mar 1;240:66-76.

119. Singh I, Hussain M, Manjunath G, Chandra N, Ravikanth G. Regenerative agriculture augments bacterial community structure for a healthier soil and agriculture. Front Agron. 2023 May 5;5:1134514. doi: 10.3389/fagro.2023.1134514. PMID: 39071943; PMCID: PMC7616306.

120. Warnasuriya SD, Udayanga D, Manamgoda DS, Biles C. Fungi as environmental bioindicators. Sci Total Environ. 2023 Sep 20;892:164583. doi: 10.1016/j.scitotenv.2023.164583. Epub 2023 Jun 3. PMID: 37277042.

121. Frąc M, Hannula SE, Bełka M, Jędryczka M. Fungal Biodiversity and Their Role in Soil Health. Front Microbiol. 2018 Apr 13;9:707. doi: 10.3389/fmicb.2018.00707. PMID: 29755421; PMCID: PMC5932366.

122. Waring BG, Averill C, Hawkes CV. Differences in fungal and bacterial physiology alter soil carbon and nitrogen cycling: insights from meta-analysis and theoretical models. Ecol Lett. 2013 Jul;16(7):887-94. doi: 10.1111/ele.12125. Epub 2013 May 22. PMID: 23692657.

123. Muturi EJ, Dunlap CA, Perry WL, Rhykerd RL. Cover crop species influences soil fungal species richness and community structure. PLoS One. 2024 Sep 12;19(9):e0308668. doi: 10.1371/journal.pone.0308668. PMID: 39264892; PMCID: PMC11392335.

124. Anand CK, Apul DS. Composting toilets as a sustainable alternative to urban sanitation--a review. Waste Manag. 2014 Feb;34(2):329-43. doi: 10.1016/j.wasman.2013.10.006. Epub 2013 Nov 21. PMID: 24268916.

125. Vázquez MA, Sen R, Soto M. Physico-chemical and biological characteristics of compost from decentralised composting programmes. Bioresour Technol. 2015 Dec;198:520-32. doi: 10.1016/j.biortech.2015.09.034. Epub 2015 Sep 18. PMID: 26432056.

126. Bernai MP, Paredes C, Sánchez-Monedero MA, Cegarra J. Maturity and stability parameters of composts prepared with a wide range of organic wastes. Bioresour Technol. 1998 Jan;63(1):91-9.

127. Rigby H, Clarke BO, Pritchard DL, Meehan B, Beshah F, Smith SR, Porter NA. A critical review of nitrogen mineralization in biosolids-amended soil, the associated fertilizer value for crop production and potential for emissions to the environment. Sci Total Environ. 2016 Jan 15;541:1310-1338. doi: 10.1016/j.scitotenv.2015.08.089. Epub 2015 Nov 11. PMID: 26476511.

128. Gale ES, Sullivan DM, Cogger CG, Bary AI, Hemphill DD, Myhre EA. Estimating plant-available nitrogen release from manures, composts, and specialty products. J Environ Qual. 2006 Oct 27;35(6):2321-32. doi: 10.2134/jeq2006.0062. PMID: 17071903.

129. Eghball B, Ginting D, Gilley JE. Residual Effects of Manure and Compost Applications on Corn Production and Soil Properties. Agron J. 2004 Mar 1;96(2):442-7.

130. Bielek K, Stinner D, McGovern S, Dickinson J, Rzewnicki P, Spray R, et al. Organic Transition Guide. Ohio: The Ohio State University and Ohio Ecological Food and Farm Association. 2015. Available from: https://www.ams.usda.gov/sites/default/files/media/Organic%20Transition%20Guide%20v3.2.pdf

131. Cioca L, Ciomoş A, Şeitoar D, Druţă RM, David GM. Industrial Symbiosis through the Use of Biosolids as Fertilizer in Romanian Agriculture. Reycl. 2021;6(3):59, DOI: 10.3390/recycling6030059

132. Lu Q, He ZL, Stofella PJ. Land Application of Biosolids in the USA: A Review. Appl Environ Soil Sci. 2012 Aug 1;2012(1):201462.

6. 7. Supporting Information

S1 – Detailed Plot Management Diary

S2 – Humanure batch collection and composition

S3 – Barley head damage and disease examples

S4 – Additional Crop Growth Graphs

S5 – Additional Soil Parameter Graphs

Figure 1 Click here to access/download;Figure;Figure 1 - Plot layour.png

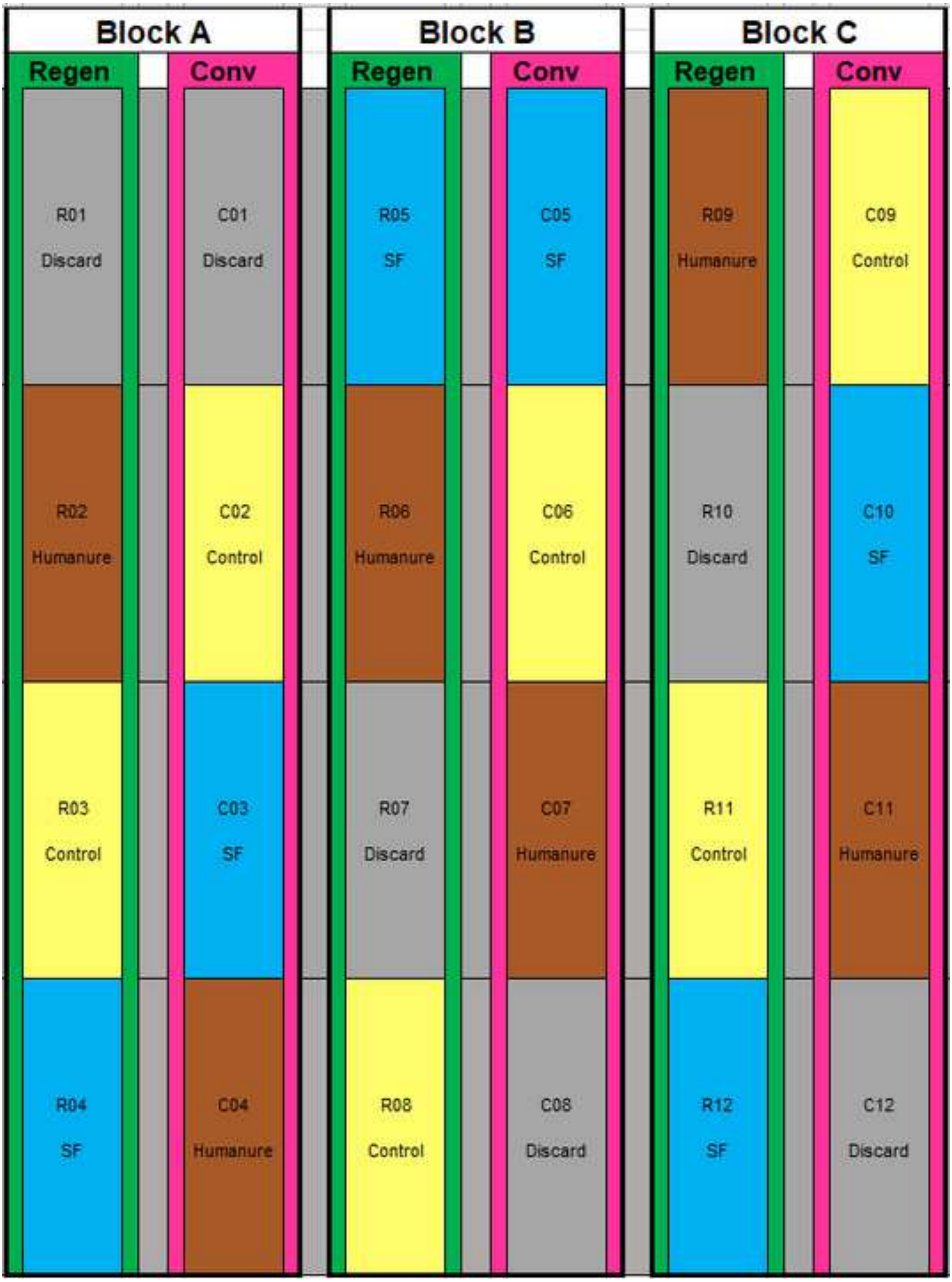

Figure 2

Click here to access/download;Figure;Figure 2 - Spring barley yield.png

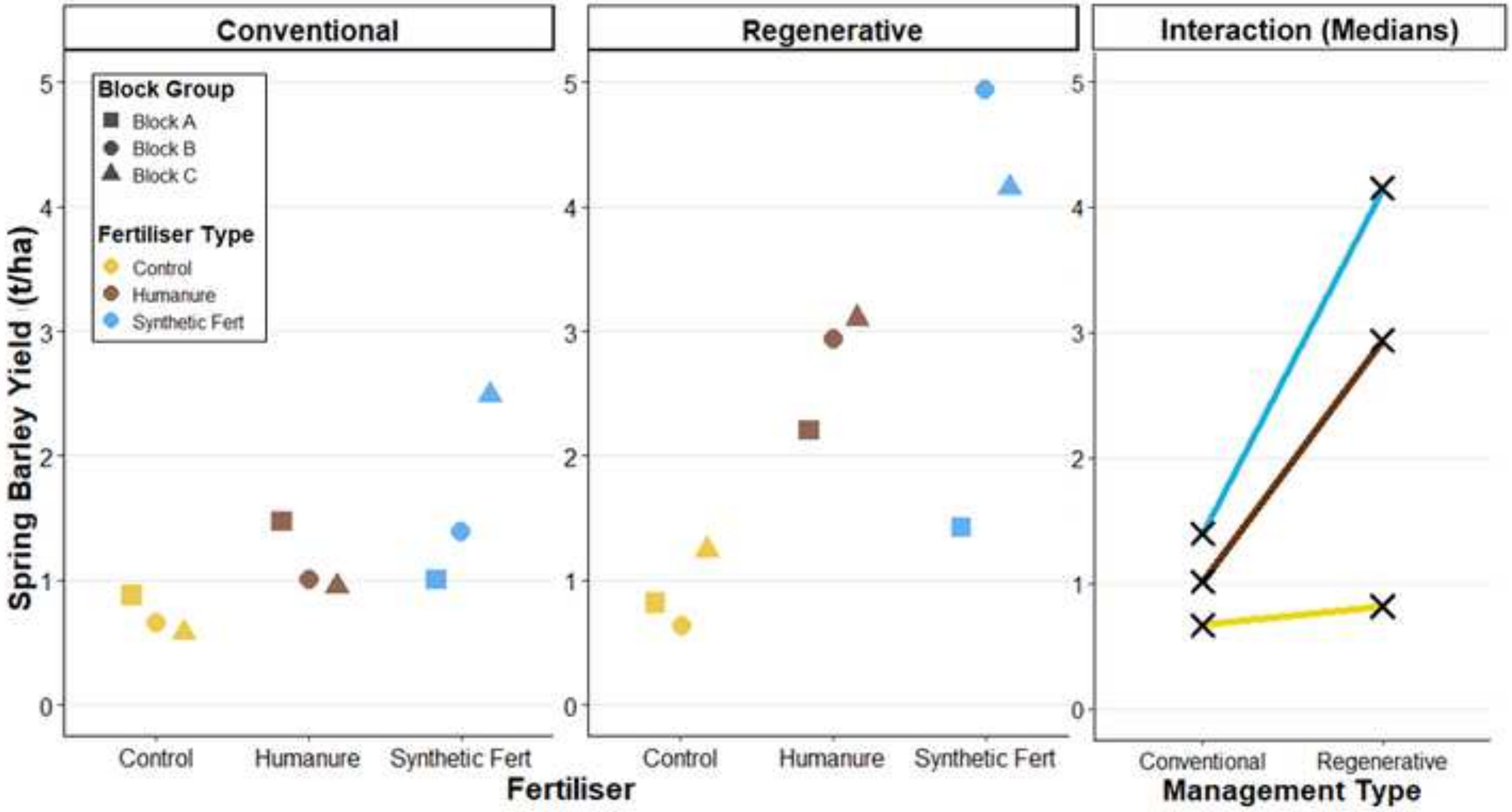

Figure 3

Click here to access/download;Figure;Figure 3 - WSA.png

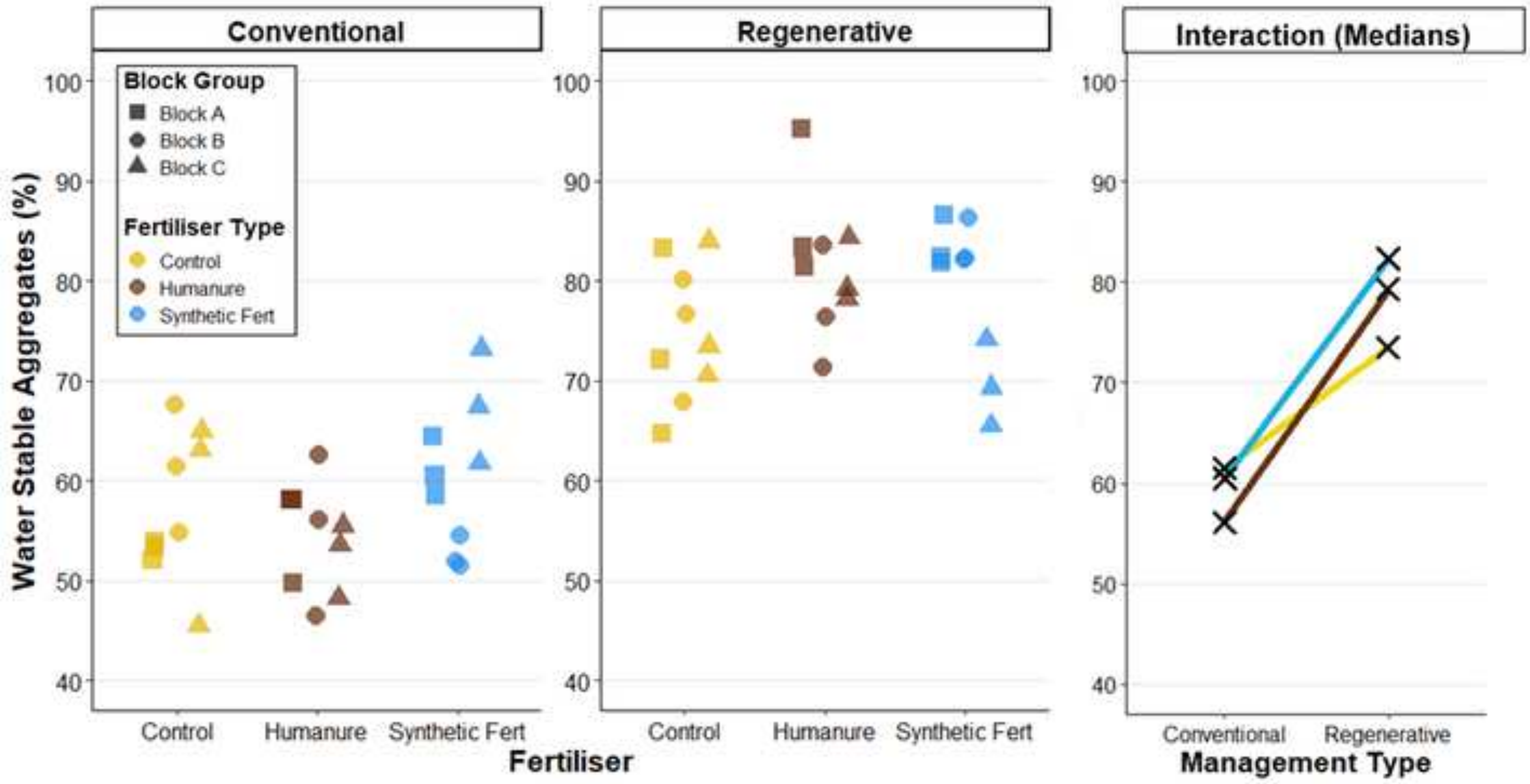

Figure 4

Click here to access/download;Figure;Figure 4 - pH.png

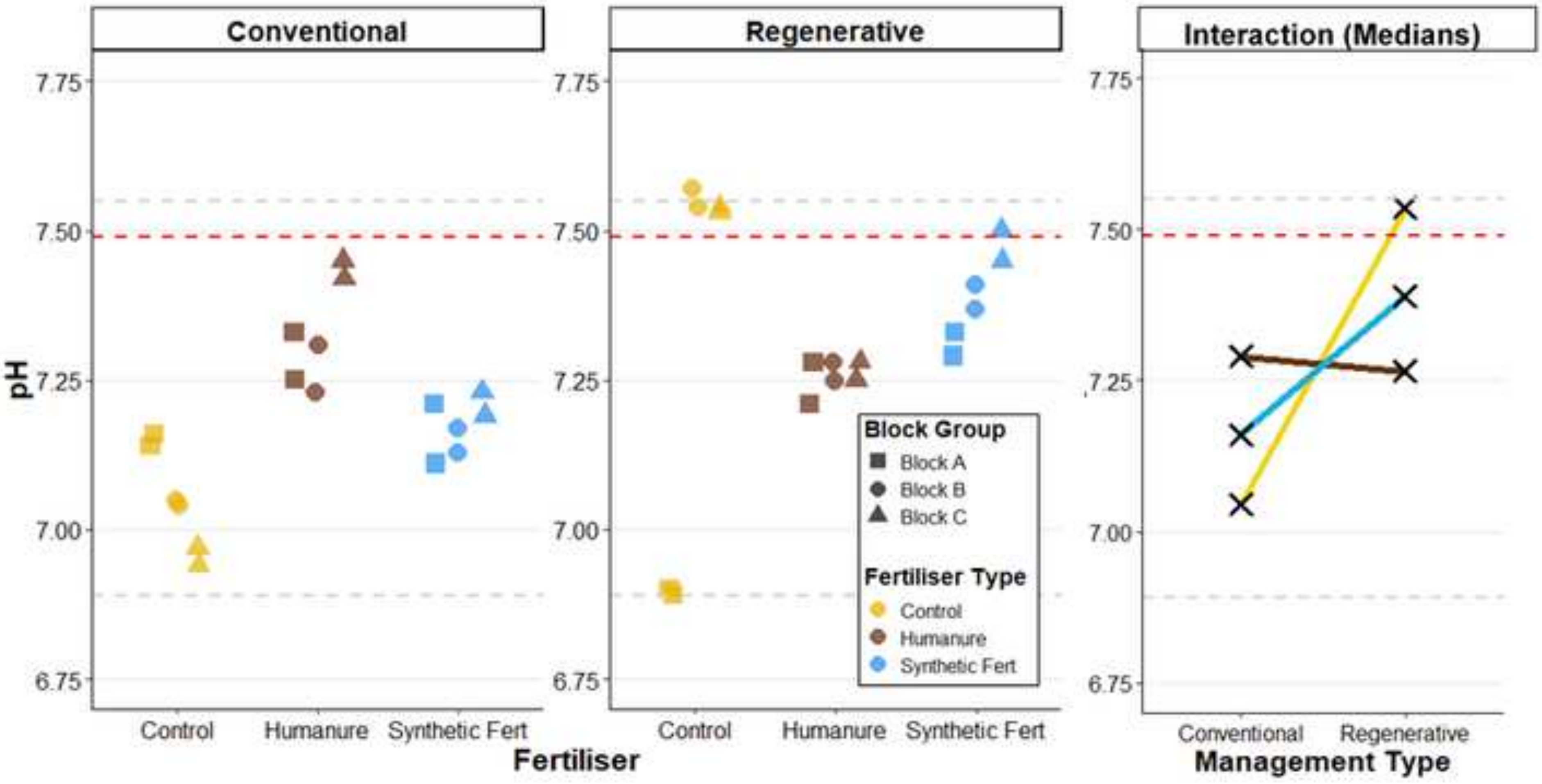

Figure 5

Click here to access/download;Figure;Figure 5 - Moisture content.png

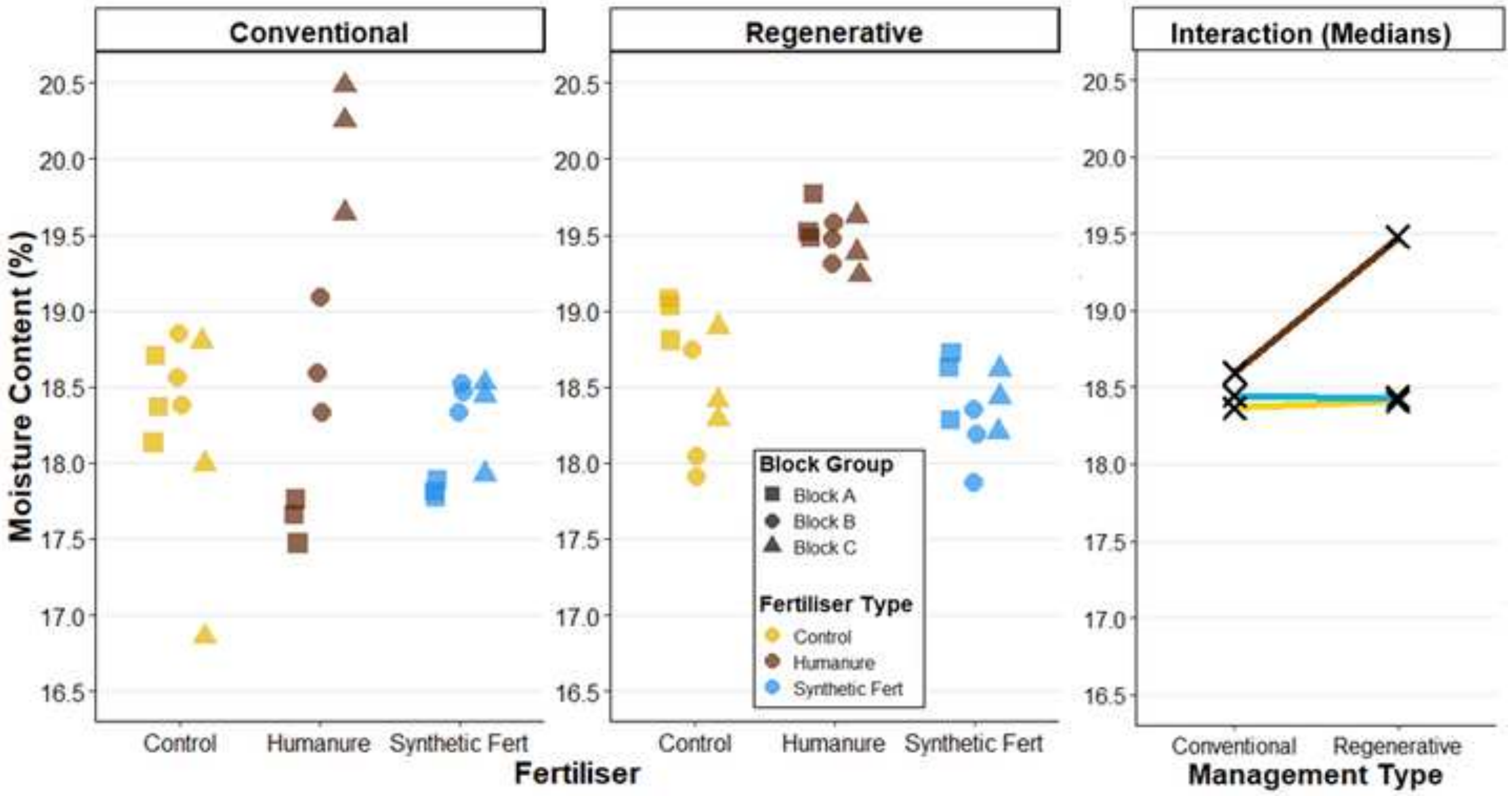

Figure 6

Click here to access/download;Figure;Figure 6 - SOM.png

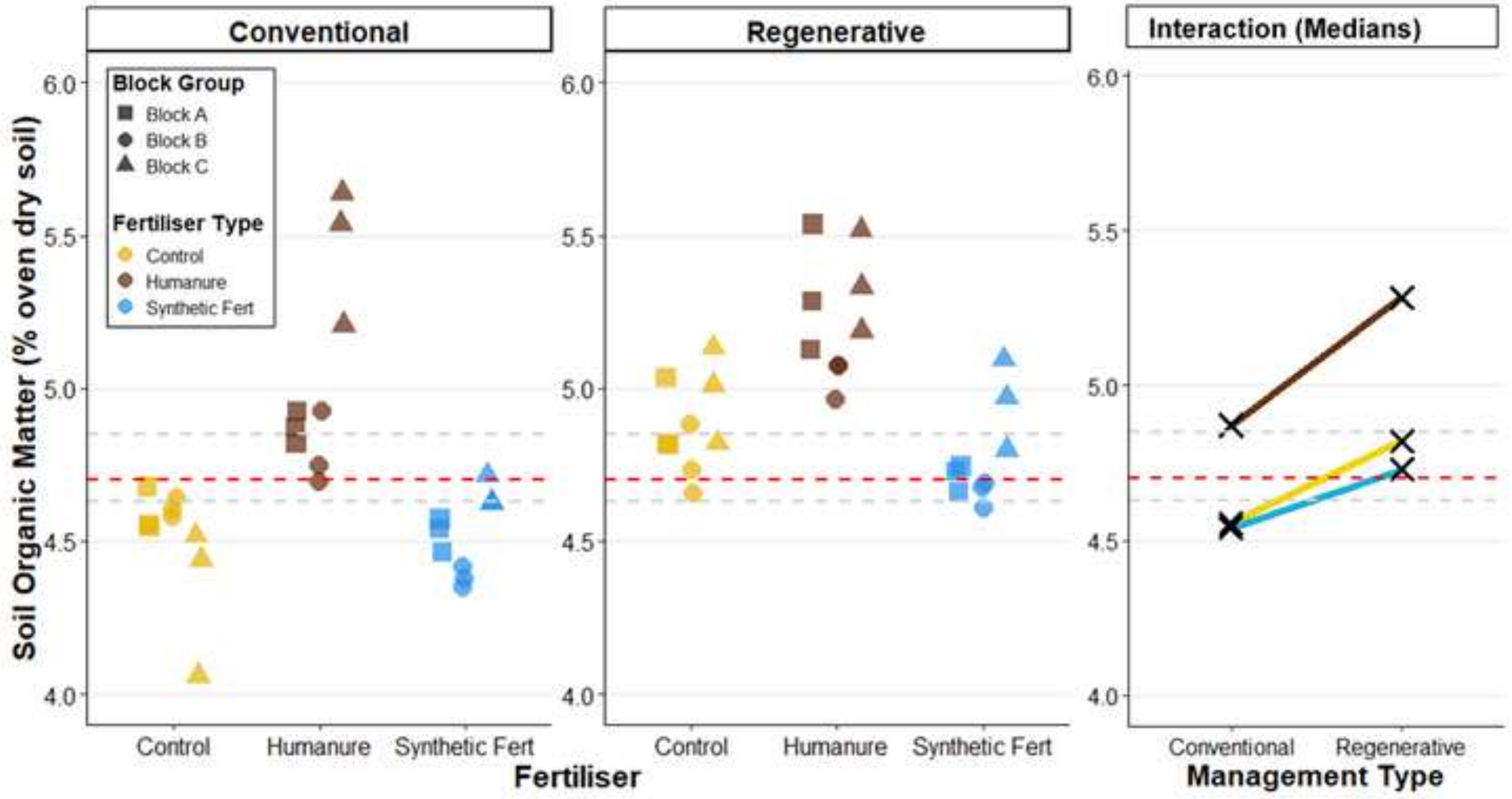

Figure 7

Click here to access/download;Figure;Figure 7 - Total N.png

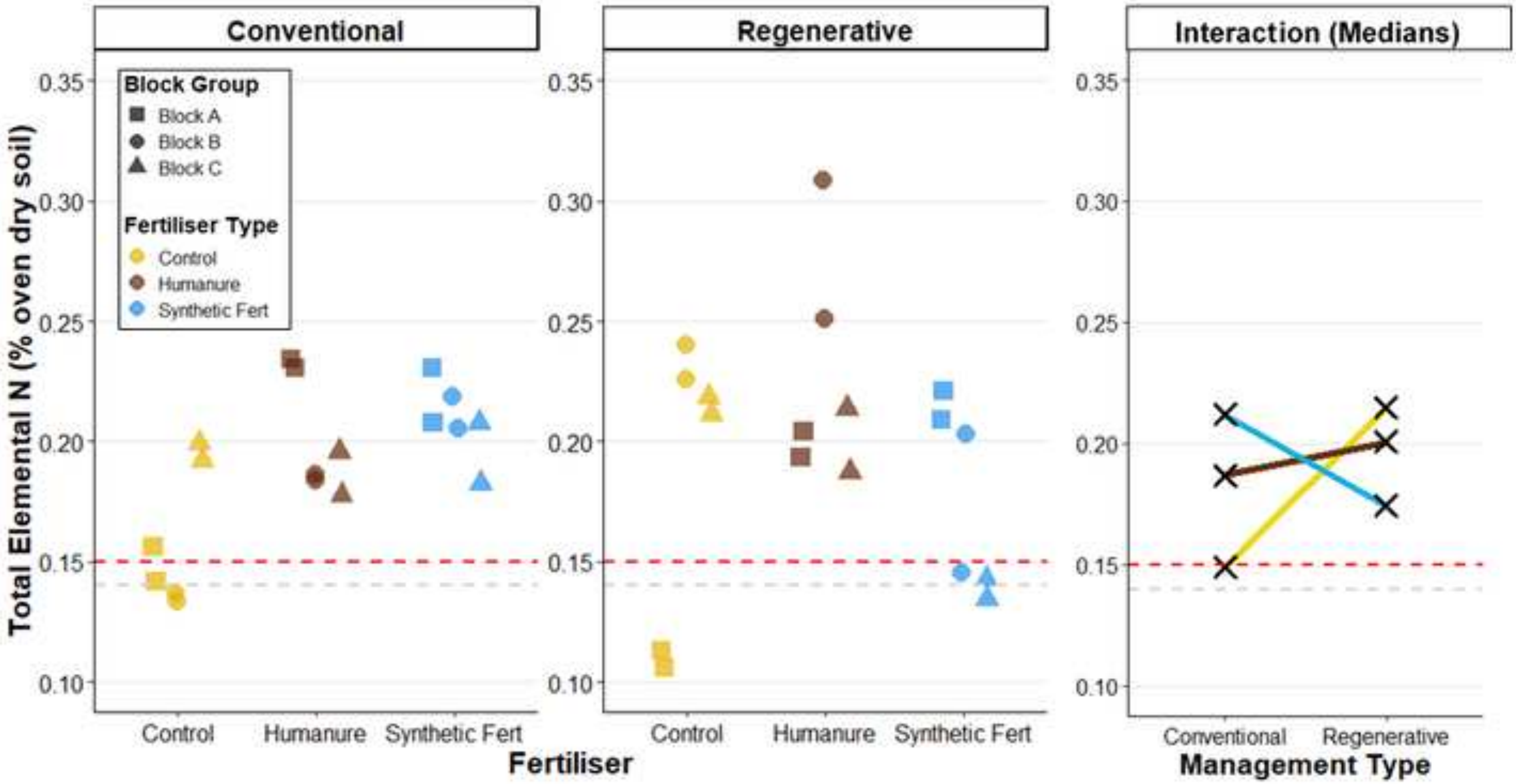

Figure 8

Click here to access/download;Figure;Figure 8 - Inorganic N.png

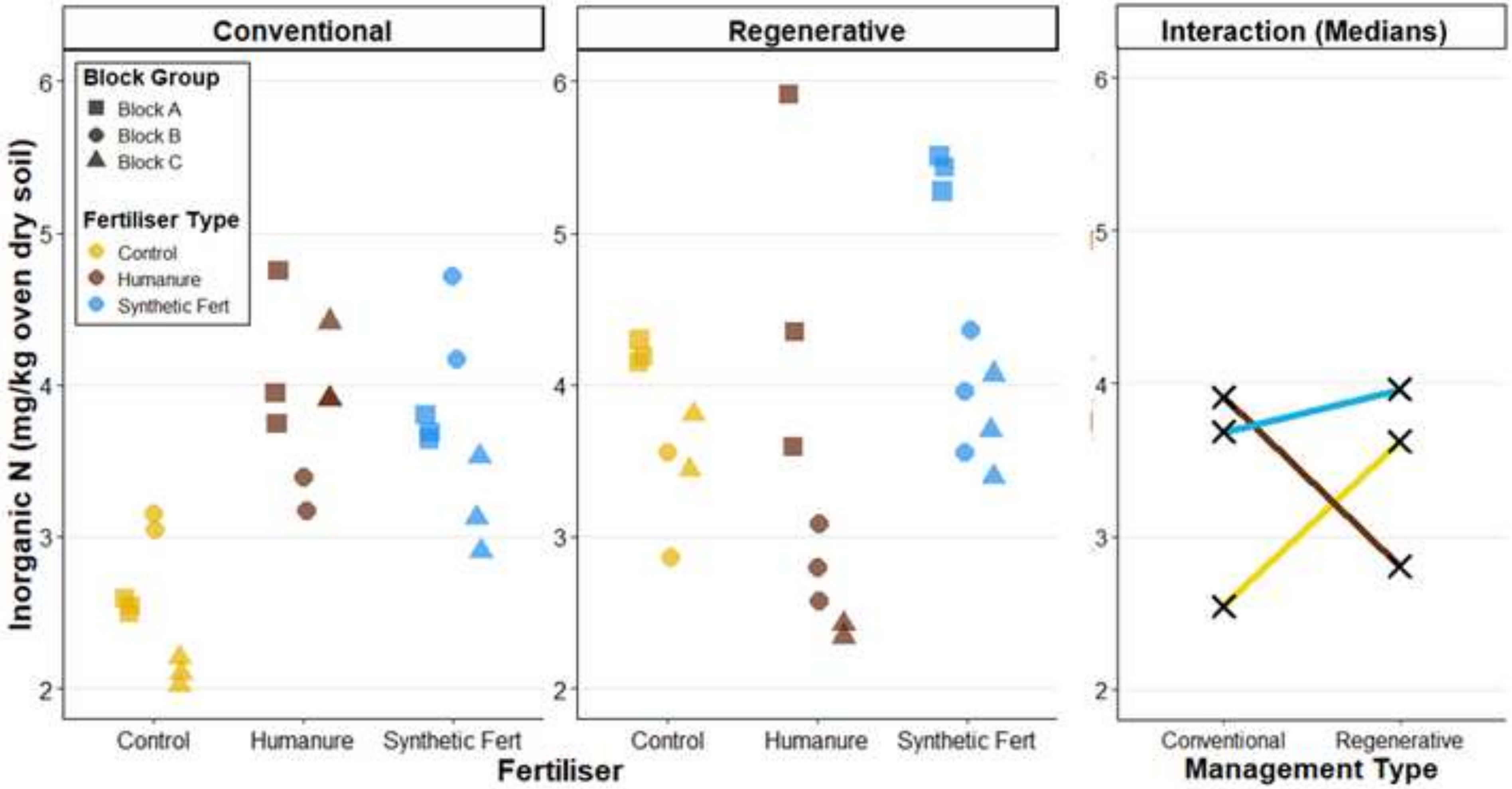

Figure 9

Click here to access/download;Figure;Figure 9 - Available P.png

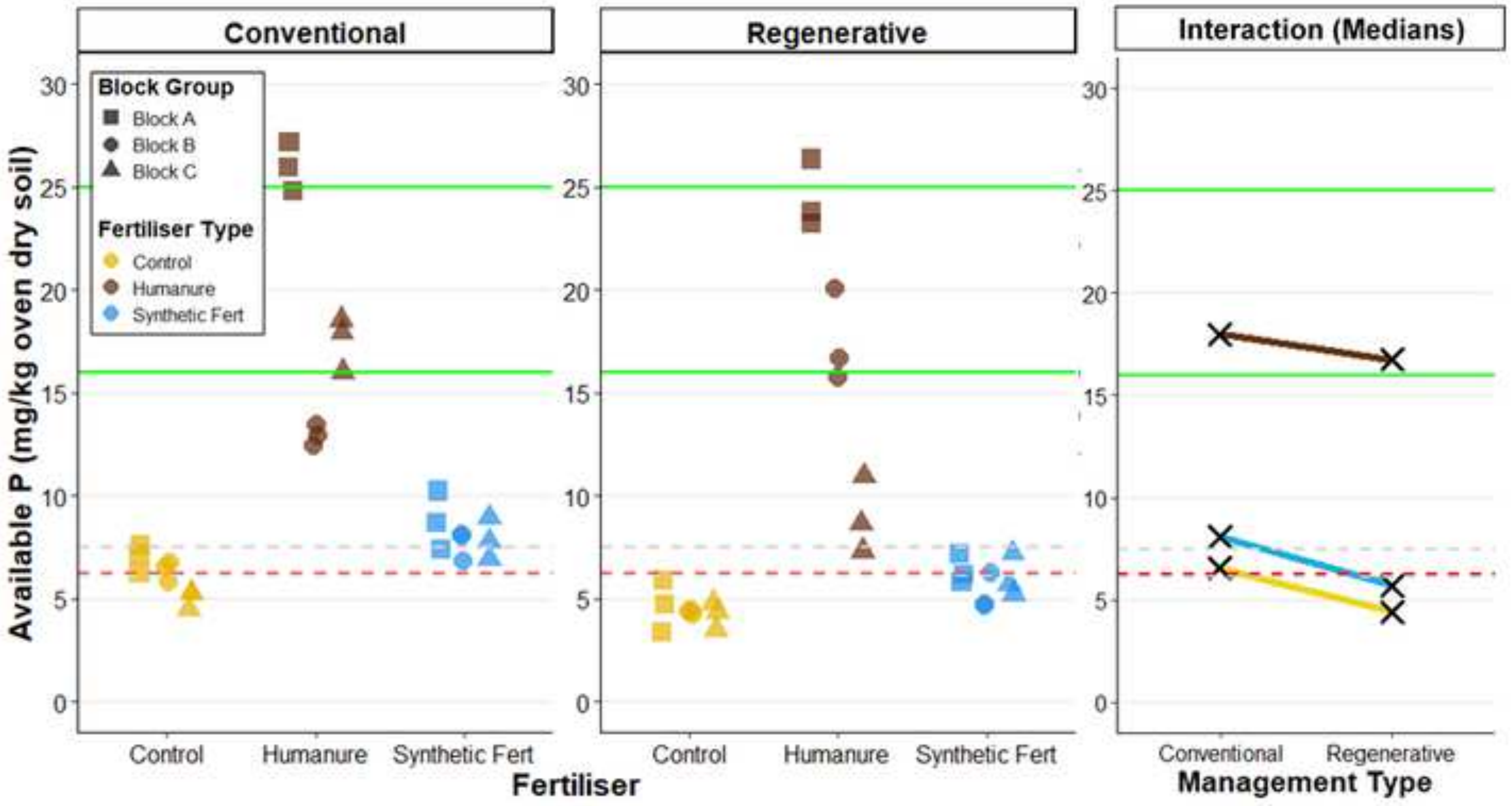

Figure 10

Click here to access/download;Figure;Figure 10 - K+.png

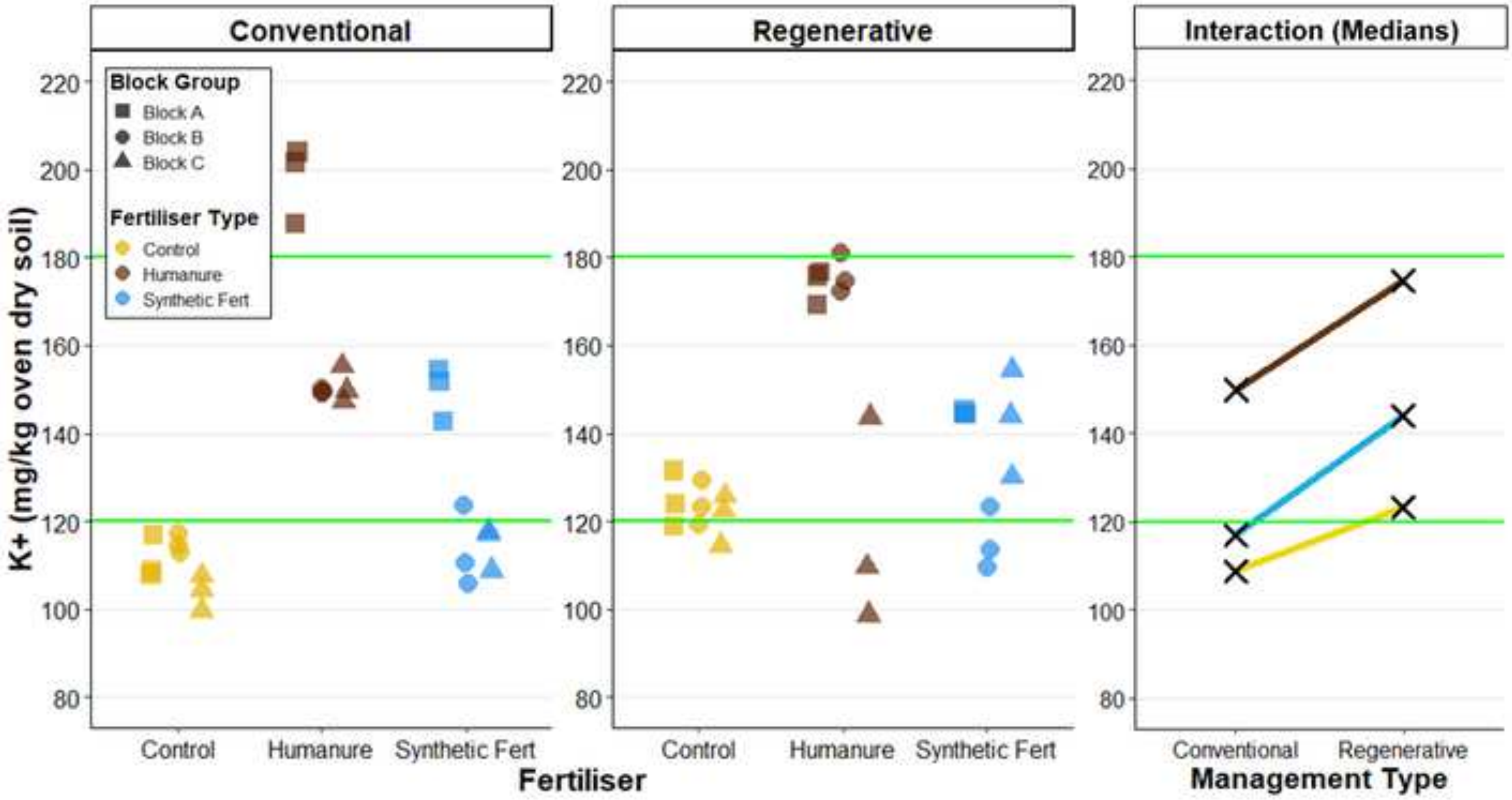

Figure 11

Click here to access/download;Figure;Figure 11 - Worms.png

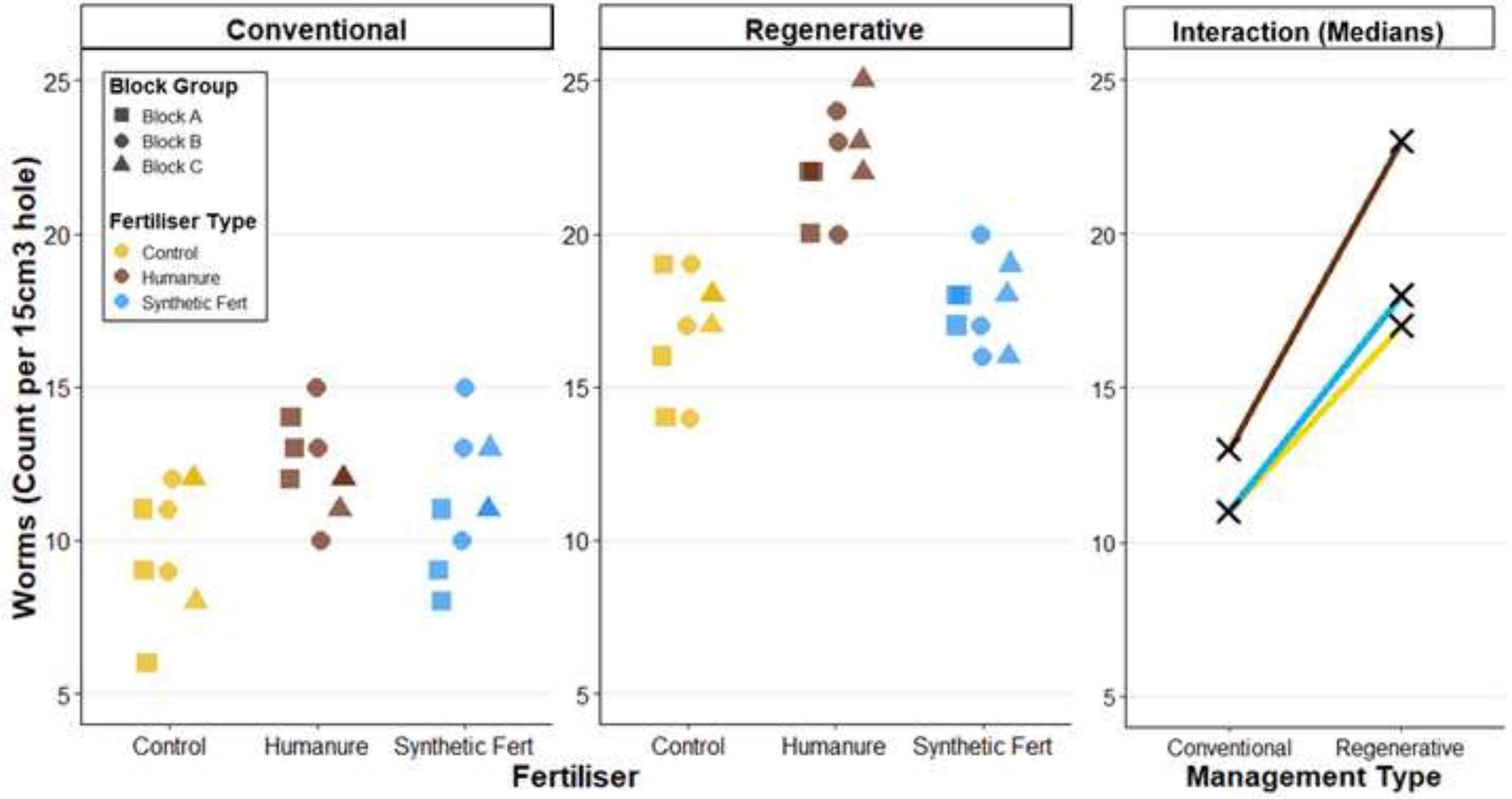

Figure 12 Click here to access/download;Figure;Figure 12 - Respiration.png

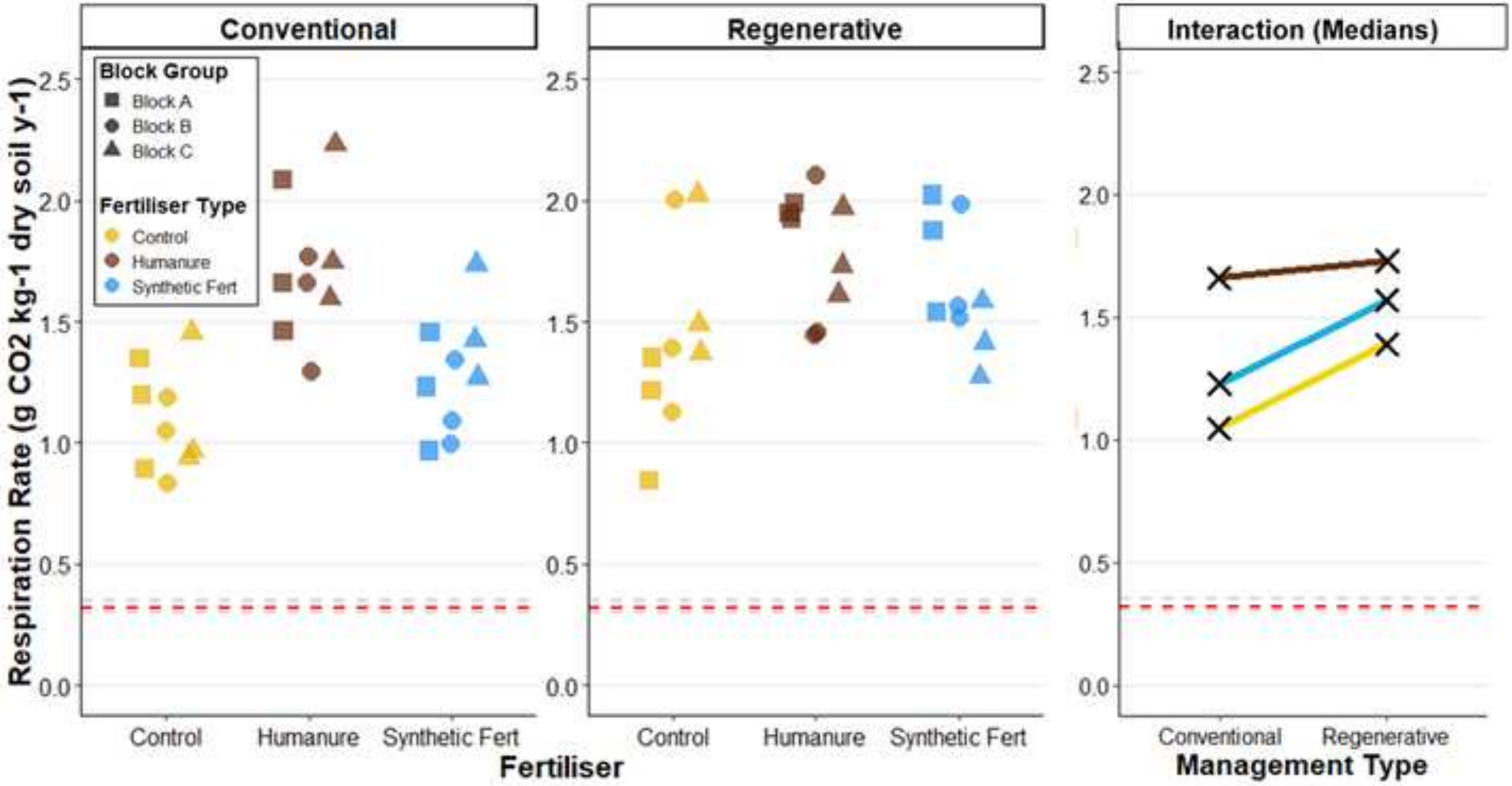

Figure 13

Click here to access/download;Figure;Figure 13 - Fungal Biomass.png

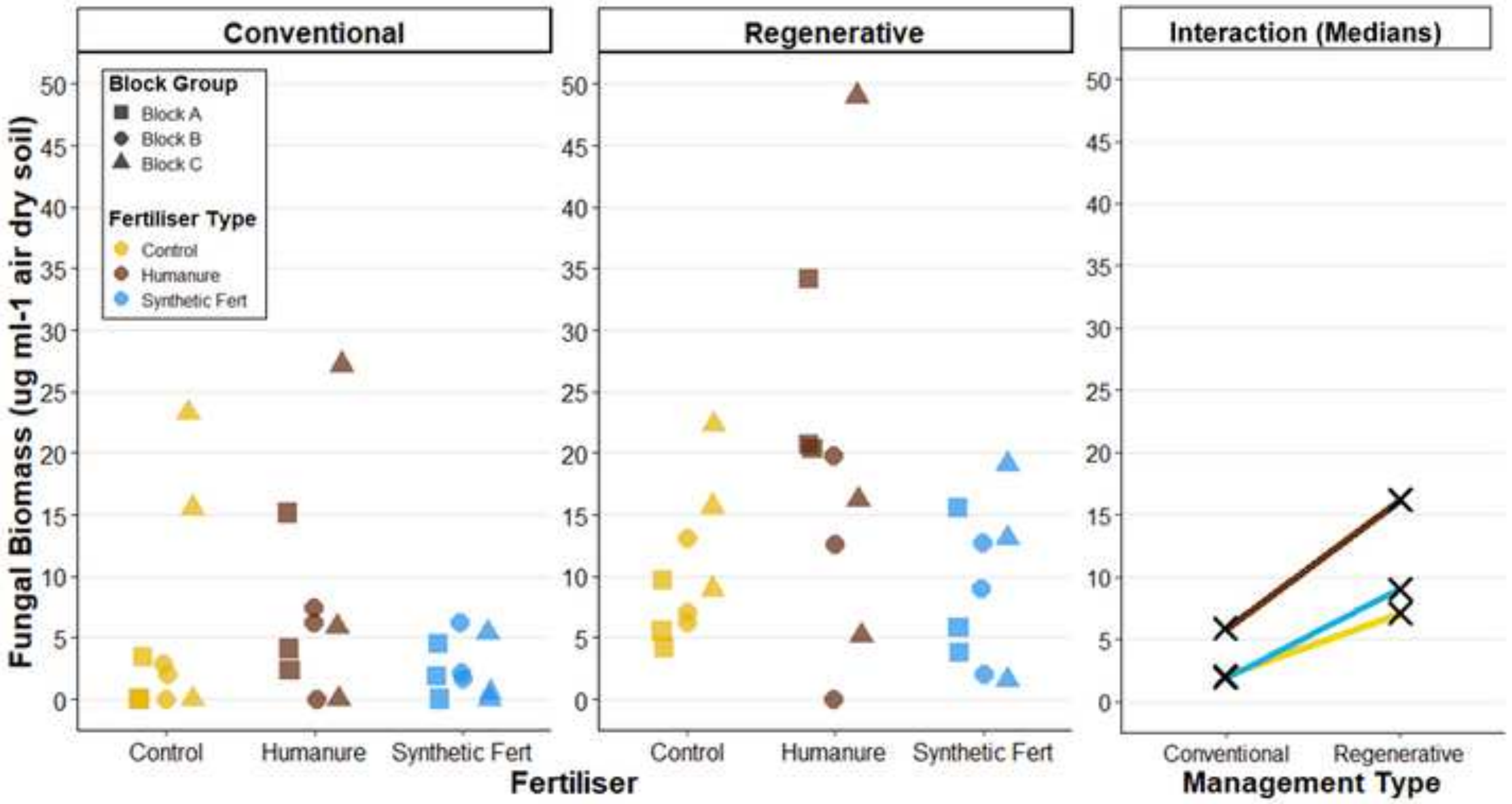

Supporting Information 1

Click here to access/download
Supporting Information
S1_File.docx

Supporting Information 2

Click here to access/download
Supporting Information
S2_File.docx

Supporting Information 3

Click here to access/download
Supporting Information
S3_File.docx

Supporting Information 4

Click here to access/download
Supporting Information
S4_File.docx

Supporting Information 5

Click here to access/download
Supporting Information
S5_File.docx
