# Supplementary material for: The use of humanure for cereal production under conventional and regenerative farming models - findings from a three-year grassland-to-arable transition
Source: PLoS One. 2026 Mar 6;21(3):e0335625. doi: 10.1371/journal.pone.0335625 (PMC12965554; doi:10.1371/journal.pone.0335625)
Supplement: Attachment — Submitted filename: Response to Reviewers.docx [file pone.0335625.s018.docx]

**Response to Reviewers 04/09/2025**

**Response to Academic Editor**

Thank you for your swift response. I have made the requested changes and reuploaded the tracked changes and clean versions of the manuscript:

- I have included a statement which indicates additional permits were not required as the study was conducted on a university-owned research site.
- I have uploaded all the raw field data as an additional file.
- I have reformatted the manuscript headings and supporting information files to match the style guides.

**Reviewer 1 Comments and Responses:**

**Abstract: This needs to be defined in some detail. The term is used throughout the paper, but it is not clear what it means.**

*The term first appears in line 83, and the next paragraph (Line 84) explains the need to include specificity when using these kinds of terms which have multiple different interpretations.*

*Section 1.2 (Line 120) provides more detail about those features which are commonly considered under the ‘regenerative’ umbrella, and explains those features which were included within this experiment.*

*A full list of these practices undertaken on the regenerative plots is given in the Methods section in Table 1.*

**Line 12: "Humanure" is human excrement (primariy fecal material and urine) recycled for agricultural purposes. It is recommended that the "toilet material" be composted prior to agricultural use. There is no evidence presented here that the humanure was composted.**

**Composting, by definition, requires the production of internal biological heat in the organic material being composted, as described in the Humanure Handbook 4th edition (2019). The 1st edition of the Humanure Handbook (1994) was used as a reference in this study.**

**The use of the word "humanure" in the title to this paper is correct. The humanure used in this study consisted of the aged contents of "dry toilets.," which  consisted of humanure, along with whatever other contents were deposited in the toilets.**

**If the toilet contents had been composted, there should be data regarding the organic materials that were utilized (humanure and what else), plus time and temperature factors. "Sawdust" was added, but was it sawdust, wood shavings, or wood chips? Was urine separated from the toilet contents, as is often the case with dry toilets?**

**Separation of urine and inclusion of large carbon chunks (wood chips), will drastically alter the nutrient composition of the organic mass. These are important factors, and this sort of information, if included, would improve this research paper.**

**To rectify this, I would suggest changing the definition of humanure as stated from "composted human feces" to something like "human excrement recycled for agricultural purposes."**

**I expect that you would find that correctly composted human toilet material, including the urine, when mixed with other organic discards in the composting process. (kitchen food, agricultural byproducts, and food/beverage industry discards), will provide better agricultural outcomes. Not that the outcomes were bad here, but just saying.**

*Thank you for this insightful comment; this nuance has been a tricky point throughout my research, especially when considering the differences communicating with agricultural experts, sanitation experts, and non-expert audiences. It is something I have discussed at great length in my thesis, and challenged the idea that humanure and composting are always synonymous, and suggest some alternative and nuanced definitions.*

*But, that’s away from the present point. I accept your comment here, that for this instance in this paper the phrasing is indeed wrong. As such I have amended it to your suggested wording, and have increased the amount of detail given for each humanure batch used in the experiment.*

**Line 192: What kind(s) of sawdust? From trees or lumber? Fresh sawdust or aged? Actual sawdust, or woodchips or wood shavings (see Humanure Handbook 4^th^ edition (2019).**

*Thank you for raising the need for more specificity here. I have updated this line to explain that the batches differed, and have also included more details about each batch in the Supporting Information 2 file. Since these materials were arriving to me second-hand, where information about the contents is unknown, this is now made more clear.*

**Line 361: Typo. should be "do"?**

*Well spotted, thank you! Fixed.*

**Line 379: typo**

*Well spotted, thank you! Fixed.*

**Line 582: Are these words necessary?**

*A valid question. They were included in attempts to move away from yield being perceived as the only metric of success, as this paper also considers the impacts on improving soil health. The sentence has been reworded for clarity.*

**Line 625: typo**

*Well spotted, thank you! Fixed.*

**Line 629: typo**

*Well spotted, thank you! Fixed.*

**Line 672: should be two words**

*Well spotted, thank you! Fixed.*

**Line 854: This is truly a ground-breaking study (pun intended). Hats off to the researchers!**

**FYI, the Glastonbury music festival utilizes what are probably “dry toilets” (as opposed to compost toilets) where a LOT of toilet material is collected and presumably composted off-site. From what I can tell, these toilets are being well-managed and could be a great source of material for future research.**

**I don’t see any major problems with your research, other than the minor issues I have mentioned in my comments. Better sources of humanure would likely make a major difference in outcomes.**

**Get your hands on the Humanure Handbook 4th edition (2019), as well as the Compost Toilet Handbook (2021). They can be downloaded as PDFs online. I can provide free PDF copies to researchers. Just email me a request (**[**joe@josephjenkins.com**](mailto:joe@josephjenkins.com)**).**

**Joe Jenkins**

*Thank you for these kind words, and for taking the time to review my research. I really appreciate it!*
